# AI-driven psychological and cognitive decision processes in professional practice: a systematic review using music teachers as an instrumental case

**DOI:** 10.3389/fpsyg.2026.1866711

**Published:** 2026-06-15

**Authors:** Yuchen Wang, Yanqing Lan

**Affiliations:** 1 The Graduate School Arts & Culture, Sangmyung University, Seoul, Republic of Korea; 2 School of Music and Dance, Yichun Early Childhood Teachers College, Yichun, Jiangxi, China

**Keywords:** AI adoption, artificial intelligence, cognitive appraisal, human–AI collaboration, music teachers, professional identity, professional judgment, psychological mechanisms

## Abstract

Artificial intelligence (AI) is increasingly entering professional practice, raising questions about how professionals interpret algorithmic authority, protect judgment autonomy, and negotiate human–AI boundaries. This systematic review uses music teachers as an instrumental case to examine AI-related psychological responses, cognitive appraisals, and professional decisions in a judgment-intensive and emotionally involved educational context. Following PRISMA 2020, 20 studies published from 2023 onwards were synthesized through thematic synthesis, directed content analysis, and higher-order evidence-to-theme mapping. MMAT 2018 was used for quality appraisal, with evidence-tiering applied to classify evidence strength. The synthesis identified four interrelated psychological–cognitive–professional decision pathways: professional boundaries and retention of judgment authority, learning-process regulation and critical engagement, adaptive co-creation and professional capacity reconstruction, and risk perception with bounded adoption. Across these pathways, music teachers did not simply accept or reject AI; rather, they selectively integrated AI by weighing technological convenience, pedagogical value, professional responsibility, and risks to student agency or cultural interpretation. A further synthesis showed that the depth and mode of AI adoption were jointly shaped by individual capability, organizational environment, and technological characteristics. The review suggests that AI training for music teachers should move beyond tool operation toward judgment-oriented preparation, including task-suitability evaluation, output review, critical-use pedagogy, and ethical guidance. For psychology-related professions, the findings should be treated as hypothesis-generating propositions rather than direct clinical recommendations, because psychological service contexts involve higher ethical responsibility, decision-making risk, and professional accountability.

## 1 Introduction

Artificial Intelligence (AI) is rapidly reshaping professional practice ecologies. For core psychological professions, such as clinical psychology, counseling, and occupational health psychology, practice heavily relies on complex cognitive judgments, ethical decision-making, and deep understanding of clients (Fiske et al., 2019). When AI systems with predictive algorithms, emotion analysis, and high-fidelity output capabilities intervene in these domains, they affect not only information processing efficiency but also practitioners’ perceptions of their professional value, sense of control, and authority in decision-making. Previous research indicates that when AI begins to mediate and restructure expert-led tasks, practitioners may experience professional identity threats, affecting confidence in core roles and generating resistance to algorithmic authority as well as anxiety over human agency (Jussupow et al., 2022; Shonhe and Min, 2025).

However, in real clinical settings, due to privacy concerns, fragile practitioner-client relationships, and high ethical requirements, it is difficult for researchers to directly observe micro-level psychological defenses and cognitive decision-making under AI influence (Miner et al., 2019). These ethical and operational barriers limit empirical investigation into how AI transforms professional psychological practice.

From the perspective of occupational psychology and professional decision-making theory, psychotherapists, counsellors, and teachers can all be understood as helping professionals whose work requires intensive emotional labor, real-time psychological judgment, and relational responsiveness in ongoing interactions (Skovholt and Trotter-Mathison, 2014). Although music teachers do not conduct clinical interventions, music teaching similarly requires nuanced judgments across artistic expression, cultural context, and learners’ psychological states, as well as continuous regulation of emotional labor in classroom interaction, performance feedback, and creative guidance. The relationship between teachers’ emotional labor and occupational strain has also been supported in previous research (Yin et al., 2019). Therefore, this review does not treat music teachers as a direct substitute sample for psychotherapists, counsellors, or psychological assessors; rather, it positions music education as an instrumental case for observing how AI enters professional judgment, identity maintenance, and boundary negotiation.

In music-related professional contexts, recent evidence also suggests that AI may reshape practitioners’ perceptions of creativity and the role of human contribution in creative processes (Ma et al., 2025). Music teachers are also not a homogeneous professional group. They differ in pedagogical aims, levels of expertise, cultural responsibilities, institutional environments, and prior exposure to digital technologies. General school music teachers may place greater emphasis on student participation, foundational musical experience, and classroom accessibility (Choi, 2023; Shin and Jung, 2024). Teachers in university, pre-service, or professionally oriented music-education contexts may give greater priority to artistic standards, professional evaluation, instructional design competence, and creative performance (Cooper, 2026; Li et al., 2025; Luo and Liang, 2025). Educators working in traditional or intercultural music education also need to address cultural authenticity, contextual interpretation, algorithmic bias, and ethical representation (Jiang, 2025; Li and Wu, 2025). Because music teachers’ work involves situated judgment, emotional responsiveness, professional discretion, and negotiation of technological boundaries, their experiences can provide theoretical clues for understanding AI involvement in judgment-intensive professional practice. However, compared with clinical psychology, counselling, or psychological assessment, music education differs substantially in ethical responsibility, decision-making risk, and professional accountability. Accordingly, this review uses the music-teacher case to generate theoretical propositions for future testing in psychology-related professions, rather than to claim direct transferability to psychological service practice.

Recent reviews of AI have largely focused on technological performance, educational efficiency, or broad applications in music-related contexts (Fei et al., 2026; Zahoor et al., 2024). A more recent learner-centered review has further shown that AI tools in music education may shape learners’ self-beliefs, cognitive agency, empowerment, and dependence (Peng et al., 2026). However, this learner-centered evidence base leaves a related but distinct question underexplored: how music teachers themselves cognitively appraise AI, protect professional judgment authority, and make pedagogical decisions when AI enters their work.

To address this gap, the present study proposes the following research questions:

RQ1: What psychological and cognitive judgments do music teachers form when interacting with AI tools, and how do these influence professional decisions?

RQ2: Which individual capability differences, organizational environment, and technological characteristics shape the depth and mode of AI adoption in music teachers’ professional practice?

## 2 Operational definitions of key psychological constructs

To clarify the psychological and cognitive processes examined in this review, several key concepts are operationally defined. First, “psychological mechanisms” refer to the response patterns formed by music teachers after AI involvement, particularly in relation to professional identity, perceived control, self-efficacy, trust, risk perception, and psychological safety. Second, “cognitive decision mechanisms” refer to teachers’ evaluative processes when determining whether, when, and how AI should be used, including judgments about task suitability, output reliability, contextual fit, potential risk, and the need for human verification. Third, “professional psychological processes” refer to the processes through which teachers preserve or reconstruct professional agency, responsibility attribution, role boundaries, and judgment authority after AI enters their pedagogical work. Fourth, “cognitive scaffolding” refers to AI support for ideation, preliminary organization, feedback, material generation, or information processing that reduces certain task demands without replacing teachers’ or students’ core judgment. Fifth, “paradoxical acceptance” refers to teachers’ recognition of AI’s pedagogical convenience and potential value while simultaneously expressing concerns about overreliance, misinformation, plagiarism, weakened originality, or reduced critical thinking. Sixth, “symbolic adoption” refers to limited, peripheral, or surface-level AI use under institutional requirements, peer norms, or low-risk experimentation, without substantially transforming core pedagogical judgment or professional practice.

Importantly, these concepts are used in this review as interpretive constructs derived from the original evidence and theoretical framework, rather than as psychological variables directly validated across all included studies.

## 3 Theoretical framework

This study examines the impact of AI on music teachers’ occupational psychology, focusing on cognitive judgment, professional decision-making, and identity management. To systematically analyze teachers’ responses under AI, we draw on social cognitive theory (SCT) and identity threat/ self-verification theory as the core theoretical framework.

### 3.1 Social cognitive theory

SCT (Bandura, 1986, 2001) posits that individual behavior is shaped by the interactive effects of self-efficacy, observational learning, and environmental feedback. In AI-assisted contexts, teachers’ self-efficacy derives both from teaching experience and their mastery of AI tools, as well as their understanding and internalization of feedback outcomes. Specifically, teachers develop confidence in professional and AI-assisted competence by operating AI, observing peer practice, and receiving immediate system or student feedback. This self-efficacy directly influences AI adoption, decision-making, and exercise of professional judgment. For example, teachers with high self-efficacy in generative AI or real-time feedback environments are more likely to delegate low-level tasks (e.g., immediate correction or harmony generation) to AI, preserving cognitive resources for higher-order judgment and creative decision-making (Li et al., 2025; Luo and Liang, 2025).

### 3.2 Identity threat and self-verification theory

Identity threat theory (Breakwell, 2015) and self-verification theory (Swann Jr and Read, 1981) provide a core lens to understand professional boundary maintenance under AI. When AI exhibits high automation in generation and evaluation, teachers may perceive threats to core professional identity (e.g., creative judgment, decision autonomy, leadership), triggering professional identity threats (Luo and Liang, 2025; Weatherly, 2026).

These ontological threats induce cognitive tension and paradoxical acceptance: teachers rationally recognize AI’s efficiency and feedback potential, yet instinctively guard against losing control over critical professional decisions (Li et al., 2025). To maintain professional self-concept and mitigate threats, teachers set psychological and behavioral boundaries, including strict review of AI-generated content, retention of ultimate aesthetic judgment, and safeguarding cultural and artistic authenticity (Li et al., 2025; Weatherly, 2026).

## 4 Method

**Figure 1:**
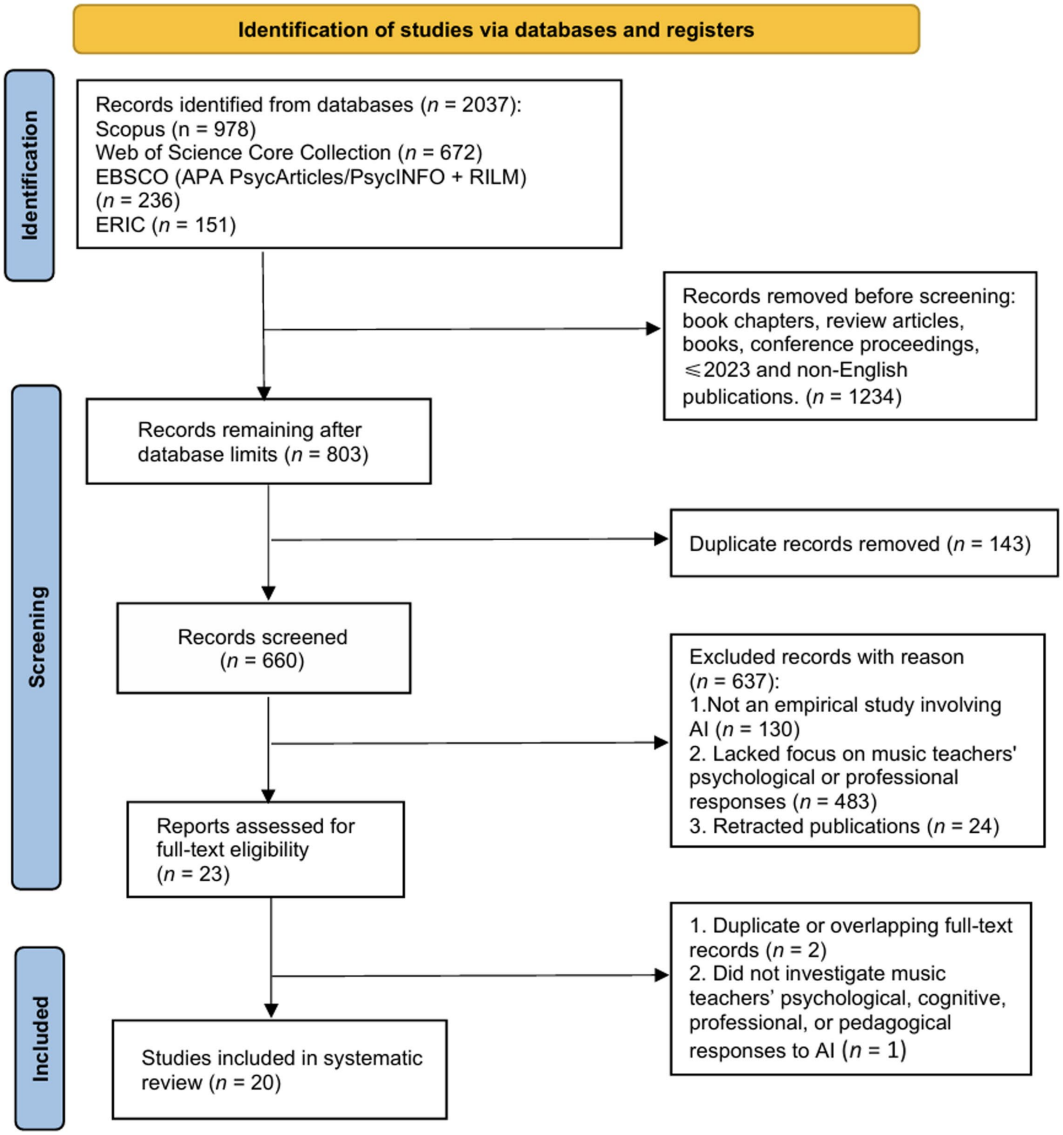
PRISMA flow diagram of study selection.

This study was conducted as a systematic literature review and organized according to the PRISMA 2020 reporting framework, including study identification, screening, eligibility assessment, and inclusion. To enhance transparency and reproducibility, each stage of the selection process is described step by step in the text, while Figure 1 reports the number of records retrieved from each database, records removed before screening, records screened by title and abstract, reports assessed for full-text eligibility, and studies finally included in the review.

### 4.1 Literature search strategy and process

The systematic search was completed on April 10, 2026, across five databases or database platforms, including Scopus, Web of Science Core Collection, ERIC, and APA PsycArticles/PsycINFO and RILM via the EBSCO platform. The search strategy was constructed around three conceptual blocks: (1) the music education domain; (2) teachers, educators, or pedagogical actors; and (3) artificial intelligence and related tools. The search syntax was adjusted according to the indexing rules of different databases. For example, Scopus used the TITLE-ABS-KEY field, Web of Science used the Topic field, and APA PsycArticles/PsycINFO and RILM used title, abstract, subject terms, or keyword fields according to the EBSCO platform rules. No additional restrictions were applied in ERIC. The complete database-specific search strategies are provided in Supplementary Table 1.

The publication year was limited to 2023 onwards. This time frame was selected because generative AI, represented by ChatGPT, rapidly entered educational and professional practice contexts after late 2022, making the influence of AI on music teachers’ pedagogical judgment, professional identity, and human–AI boundaries a new research issue. Therefore, records published before 2023 were removed before screening.

The screening process consisted of four stages. First, at the identification stage, 2,037 records were retrieved. Second, before screening, 1,234 records were removed based on record metadata because they involved ineligible publication types, non-English publications, or records published before 2023; 143 duplicate records were then removed, leaving 660 records for title and abstract screening. Third, at the title/abstract and metadata screening stage, 637 records were excluded. Fourth, 23 reports were assessed for full-text eligibility, of which two were excluded because of duplicate or substantially overlapping full texts, and one was excluded because it did not address music teachers’ psychological, cognitive, professional, or pedagogical responses to AI. Finally, 20 studies were included in the systematic review. The detailed inclusion and exclusion criteria are presented in Table 1.

**Table 1:** Inclusion and exclusion criteria.

Domain	Inclusion criteria	Exclusion criteria
Population	Articles involving music teachers, music educators, pre-service music teachers, music tutors, music teaching facilitators, or actors involved in music curriculum implementation.	Studies whose full text did not involve music teachers, music educators, or relevant teaching actors at all.
Phenomenon/intervention	Studies involving artificial intelligence, generative AI, AI-assisted teaching tools, automated feedback systems, intelligent tutoring systems, AI-based music generation, AI-assisted assessment, or AI-mediated music teaching practice.	Studies that only evaluated AI system performance, algorithmic accuracy, or technical development without addressing teachers’ use, judgment, or responses.
Context	Studies conducted in educational or teaching contexts within a music-related setting.	Non-music education contexts.
Focus of inquiry	Studies addressing at least one of the following: music teachers’ psychological responses, professional identity, cognitive appraisal, pedagogical judgment, or intention to adopt AI.	Studies that only described AI tools or educational technology use without addressing teachers’ psychological, cognitive, professional, identity-related, or pedagogical decision-making responses.
Study type	Empirical studies, including quantitative, qualitative, mixed-methods, intervention, survey, interview, case study, design-based, and observational studies. Theoretical studies directly analyzing teachers’ professional judgment, identity, or boundary issues in AI-mediated music education could be included as supplementary analytic material.	Reviews, books, book chapters, conference papers, editorials, commentaries, opinion papers, non-academic reports, preprints, and grey literature, unless they provided direct and sufficient analytic value for the research questions.
Publication type	Formally published peer-reviewed journal articles or academic journal articles.	Books, book chapters, conference proceedings, dissertations, non-peer-reviewed reports, preprints, and other grey literature.
Publication year	Studies published from 2023 onwards.	Studies published before 2023.
Language	English-language publications.	Non-English publications.
Full-text availability	Full text was available for eligibility assessment and data extraction.	Full text was unavailable or provided insufficient information for coding and synthesis.
Retraction status	Formally published studies that had not been retracted.	Retracted publications.
Duplicate handling	When multiple records reported the same study, the most complete, most recent, or most suitable version for data extraction was retained.	Duplicate records, duplicate full texts, or substantially overlapping reports.

To ensure the objectivity and rigor of the literature screening process, inter-rater reliability was further assessed. The first author first independently completed the preliminary review and screening of the full sample. Subsequently, another researcher with relevant professional expertise randomly selected 20% of the records for a second independent check, covering both the title/abstract screening stage and the full-text assessment stage. Agreement between the two reviewers was high, with Cohen’s *κ* = 0.92. Any ambiguities or inconsistencies that emerged during screening were resolved by rechecking the original articles and discussing them until consensus was reached, thereby ensuring the accuracy and reliability of the final list of included studies.

### 4.2 Data extraction and synthesis

This study combined thematic synthesis with directed content analysis to extract and synthesize data from the 20 included studies. First, basic identification information was extracted for each study, including study code, country/context, participants, research design and methods, AI tool or use context, and key findings; these details are presented in Supplementary Table 2. To enhance the transparency of the synthesis process, a comprehensive coding matrix was further developed to integrate study identification information with evidence-to-theme mapping, showing the correspondence between each study, the research questions, thematic pathways, and original textual evidence; this matrix is presented in Supplementary Table 3.

First, original evidence extraction and evidence-basis annotation. For RQ1, this study extracted original findings, participant quotations, study variables, and author-reported results related to teachers’ AI-related cognitive appraisals, psychological orientations, and professional responses. The first category concerned teachers’ cognitive appraisals of AI, referring mainly to how teachers understood the functions, risks, boundaries, and value of AI in teaching and professional practice. The second category concerned teachers’ psychological or attitudinal orientations, referring mainly to psychological responses such as acceptance, concern, caution, resistance, active adaptation, or normative compliance when facing AI tools. The third category concerned teachers’ professional responses and decisions, referring mainly to how teachers translated these cognitive appraisals and psychological orientations into concrete teaching behaviors, professional judgments, use restrictions, co-creative practices, or institutional responses.

For RQ2, this study extracted three types of conditional factors influencing the depth and mode of AI adoption. The first category was individual capability differences, referring mainly to teachers’ own characteristics and AI readiness. The second category was organizational environment, referring mainly to policy requirements, peer norms, assessment systems, academic integrity rules, institutional support, training resources, and curriculum structures within teachers’ educational settings. The third category was technological characteristics, referring mainly to the generative capacity, degree of automation, ease of use, accuracy, black-box nature, bias risk, feedback frequency, system latency, stability, and cross-modal collaborative capacity of AI tools or platforms. At the same time, this study also extracted evidence on how these factors influenced the depth and mode of teachers’ AI adoption.

In addition, during original evidence extraction, each evidence unit was annotated according to its evidentiary basis in order to distinguish its level of empirical support. The first type consisted of constructs directly measured or explicitly reported in the original studies. The second type consisted of phenomena directly described in the original studies, such as teachers’ concerns that students might stop thinking, passively imitate, or over-rely on AI, or teachers’ emphasis that AI outputs required human review, teacher interpretation, and final judgment. The third type consisted of interpretive constructs inferred by the review authors based on the original evidence and theoretical framework.

Second, descriptive categorization was used to extract preliminary raw features and concepts (Braun and Clarke, 2006), followed by systematic clustering based on semantic similarity and logical association. First, relevant expressions from the original studies were retained as much as possible to avoid directly imposing higher-order psychological concepts at the initial stage. Subsequently, according to the meaning, function, and decision logic indicated by different evidence units, similar original evidence was grouped into descriptive codes. The role of descriptive codes was to establish an intermediate layer between original evidence and interpretive themes, rather than directly forming final theoretical conclusions.

Third, a higher-order mapping method was used for synthesis (Booth et al., 2021). For RQ1, the researchers further examined whether different descriptive codes pointed to similar teachers’ cognitive judgments, psychological orientations, and professional responses, and then conducted higher-order induction based on their shared decision logic. This process did not simply count the frequency of a given theme; rather, it focused on whether different pieces of evidence formed an interpretable pattern, namely how teachers understood AI, how they formed corresponding psychological or attitudinal responses, and how these responses were further transformed into professional judgments or pedagogical decisions. The division of different pathways does not imply that these psychological–cognitive processes are completely independent of one another. Instead, the pathways were categorized according to the core judgment issue indicated by each piece of evidence, inclusion boundaries, exclusion boundaries, core pathway concepts, and primary and secondary supporting studies. The specific pathway boundaries and decision criteria are presented in Supplementary Table 4.

For RQ2, the researchers further examined how different descriptive codes reflected the conditional factors regulating the depth and mode of AI adoption and categorized them into three dimensions: individual capability differences, organizational environment, and technological characteristics. This classification used Bandura’s triadic reciprocal determinism in social cognitive theory as a sensitizing framework to support understanding of how individual, environmental, and technological conditions jointly shape teachers’ AI adoption behavior, rather than as a predetermined model imposed directly on the original literature. In other words, this study did not assume *a priori* that these three types of factors must necessarily operate; instead, by comparing empirical evidence across the included studies, it identified which individual capabilities, organizational environments, and technological characteristics influenced the depth and mode of AI adoption among professionals in practice.

Finally, data extraction and initial coding were first completed by the first author. Subsequently, a second researcher reviewed the data extraction table, evidence-basis annotations, descriptive codes, and higher-order thematic classifications. For coding units involving disagreement, the researchers rechecked the original evidence and discussed the most appropriate classification with reference to the evidence-to-theme matrix. If a given evidence unit supported multiple themes at the same time, the dominant classification was determined primarily according to the original study’s core research question, emphasis in the results, and strength of evidence, while secondary theme labels were retained in the matrix. All disagreements were resolved through discussion until consensus was reached.

### 4.3 Quality appraisal

This study used the Mixed Methods Appraisal Tool (MMAT) 2018 to assess the methodological quality of the included studies that reported first-hand empirical data (Hong et al., 2018). Because the studies included in this review were methodologically diverse, including qualitative interviews, questionnaire surveys, structural equation modelling, classroom interventions, design-based studies, and mixed-methods studies, MMAT 2018 was suitable for assessing the methodological quality of different types of empirical studies.

For each empirical study, this study assigned only one main MMAT category rather than assigning multiple categories simultaneously. The category judgment was based on the dominant form of evidence used by the study to answer its main research question. Specifically, studies mainly relying on interviews, observations, reflective texts, or thematic analysis were classified as Qualitative; studies mainly relying on questionnaires, ratings, SEM, PLS-SEM, or other statistical models were classified as Quantitative descriptive; studies mainly relying on non-randomized interventions, experimental/control groups, or pre-post effect comparisons were classified as Quantitative non-randomized; if both quantitative and qualitative evidence constituted core results and were integrated and interpreted in the study, the study was classified as Mixed methods. Features such as design-based, classroom case, or platform evaluation were not treated as separate MMAT categories.

The quality appraisal first judged two screening questions: first, whether the research question was clear; and second, whether the collected data could answer the research question. Then, the corresponding MMAT module was selected according to the study design type. Each module contains five core appraisal items, which were marked as “Yes,” “No,” or “Cannot tell.” Following the MMAT user guidance, this study did not use the number of “Yes” items as a formal total score, nor did it exclude studies based on quality appraisal results. To facilitate interpretation of the strength of evidence supporting the synthesis results across studies, this study only used the number of “Yes” items, the proportion of “Cannot tell,” the fit between study design and research question, and whether there were key methodological flaws as descriptive bases to form an overall quality judgment. The specific judgment criteria are shown in Table 2.

**Table 2:** Overall judgment criteria for MMAT quality appraisal.

Overall judgment	Simplified judgment criteria
High	At least 4 of Q1–Q5 were rated as Yes, with no obvious key flaw
Moderate-to-high	3 of Q1–Q5 were rated as Yes, the remaining items were mostly Cannot tell, and there was no serious methodological problem
Moderate	2–3 of Q1–Q5 were rated as Yes, but several limitations existed
Low-to-moderate	Approximately 2 of Q1–Q5 were rated as Yes, and multiple items were rated as Cannot tell
Low	0–1 of Q1–Q5 were rated as Yes, or the data could not sufficiently answer the research question

The quality appraisal results were mainly used to explain the strength of support provided by different studies for the interpretive pathways and conditional factors, rather than to exclude studies. For studies with higher methodological quality or more direct evidence, their findings had stronger empirical support in pathway generation; for studies with insufficient methodological information, more indirect evidence, or limited teacher-related data, their findings were mainly used as auxiliary evidence, practical implications, or contextual explanations.

In addition, some included literature belonged to philosophical inquiry, conceptual papers, theoretical syntheses, or system design articles and was not suitable for MMAT scoring. To avoid assigning the same evidentiary weight to different types of literature, this study further established an Evidence tier, shown in Table 3, to provide a layered explanation of the evidentiary function of all included studies. This layering was not intended to evaluate the academic value of the articles themselves, but to distinguish the strength of their empirical support for pathway generation.

**Table 3:** Evidence tier of included studies.

Tier	Strength of evidence	Literature type	Role in the review	Included studies
Tier 1	Strongest empirical support	Direct teacher/pre-service teacher data, such as interviews, questionnaires, SEM, mixed methods, and teacher ratings	Can serve as the main basis for pathway generation	S2, S5, S7, S8, S9, S11, S13, S14, S16, S17, S18
Tier 2	Moderate empirical support	Interventions, design-based studies, platform evaluations, classroom cases, and system application studies	Supports pedagogical responses, adoption modes, and design implications, but contextual limitations should be noted	S4, S6, S10, S15, S19, S20
Tier 3	Mainly conceptual support	Philosophical inquiry, conceptual papers, theoretical frameworks, or system design articles lacking first-hand teacher data	Used for conceptual interpretation, theoretical extension, and boundary discussion, but not as strong empirical evidence	S1, S3, S12

## 5 Results

### 5.1 Descriptive results

**Figure 2:**
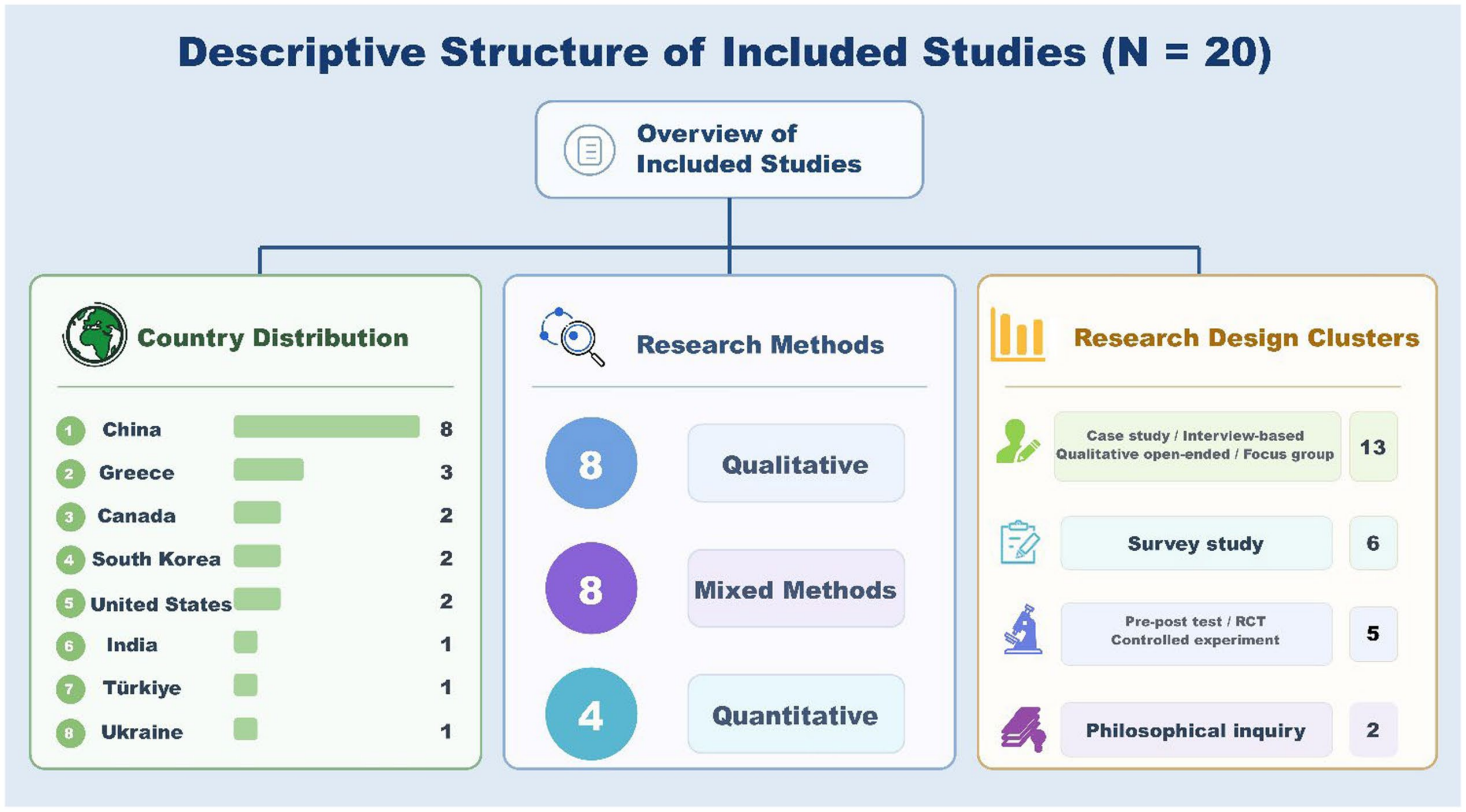
Descriptive characteristics of the included studies.

The 20 included studies demonstrated marked geographical dispersion and methodological heterogeneity. In terms of country and region distribution, the studies were primarily situated in China (*n* = 8), Greece (*n* = 3), Canada (*n* = 2), South Korea (*n* = 2), the United States (*n* = 2), and India (*n* = 1), with additional contributions from Turkey (*n* = 1) and Ukraine (*n* = 1) (see Figure 2). Overall, existing evidence is concentrated in Asia and Europe, while research in other regions remains relatively scarce.

Regarding research methods, qualitative studies and mixed-methods studies were equal in number, each comprising eight studies, while quantitative studies accounted for four, indicating that the field remains largely exploratory. In terms of study design, qualitative approaches—such as case studies, interviews, open-ended feedback, and focus groups—were most common. Survey research, pre-post designs, experimental, and intervention studies were also represented, whereas philosophical inquiry and design-based studies were relatively few. Overall, this pattern suggests the field is still in a developmental exploratory phase, showing strong context-dependence and considerable methodological diversity.

In addition, the MMAT 2018 appraisal of 17 studies showed that the overall methodological quality was moderate to high. Specifically, 7 studies were rated as High, 5 as Moderate-to-high, 3 as Moderate, and 2 as Low-to-moderate, with no study rated as Low (see Supplementary Table 5).

The main limitations were concentrated in three areas. First, some quantitative studies provided insufficient information on sample representativeness and nonresponse bias, such as S8, S11, S13, and S18, which limited the generalizability and causal interpretability of their findings. Second, although some mixed-methods studies used questionnaires, interviews, classroom interventions, or text analysis, the integration between quantitative and qualitative results was not always clear, as in S2, S4, S5, and S10. Third, in some platform evaluation, classroom case, or system application studies, evidence on teachers’ psychological and professional decision-making processes was relatively indirect, such as S10, S19, and S20. These studies were therefore more suitable as sources of practical implications, technological conditions, or auxiliary evidence, rather than as strong evidence for core psychological mechanisms. Studies with relatively lower quality included S10 and S20, both of which were rated as Low-to-moderate. Therefore, these two studies were mainly used as auxiliary evidence, and their findings should be interpreted with caution.

### 5.2 Psychological–cognitive–decision pathways of AI adoption

Based on the evidence from the included studies, the psychological cognition and professional decision-making of music teachers when engaging with AI tools can be objectively summarized into four dynamic dimensions (see Supplementary Table 4).

First, in terms of professional boundaries and retention of judgment authority, teachers cognitively recognized the auxiliary value of AI in ideation support, preliminary processing, and cognitive scaffolding, but remained cautious about its fit with professional contexts, factual accuracy, and content coherence. Accordingly, their professional decisions were often characterized by “review-based acceptance,” namely maintaining human final verification authority and relying on their own professional experience to reject outputs that did not meet disciplinary standards in music or fit the teaching context (S1, S13, S16).

Second, in terms of learning-process regulation and critical engagement, teachers showed “paradoxical acceptance.” On the one hand, they acknowledged the convenience of AI for creative inspiration, material generation, and pedagogical support; on the other hand, they were concerned that students might develop cognitive dependence, engage in plagiarism, be affected by misinformation, or weaken their capacity for original thinking (S9, S14). This educational value trade-off led teachers to actively regulate the timing of AI intervention, set rules for use, and guide students to examine generated content critically, rather than allowing AI to directly replace core thinking and creative processes (S4, S9, S14).

Third, in terms of adaptive co-creation and professional capacity reconstruction, some teachers and pre-service teachers began to understand AI not merely as an efficiency tool, but as a resource for creative generation, inclusive expression, and professional learning. The included studies indicate that AI can lower the threshold for music participation and, in training contexts, enhance teachers’ creative self-efficacy and willingness to experiment (S3, S6). In addition, evidence related to technology-impact awareness and proactive personality suggests that the impact of AI may also stimulate teachers’ active learning intention (S18). At the level of concrete practice, pre-service teachers with higher personal innovativeness were more likely to design AI-interactive elements in lesson plans; pre-service teachers who received GenAI-TPACK intervention were also better able to use AI for ideating, evaluating, and revising instructional designs (S5, S16).

Finally, in terms of risk perception and bounded adoption, teachers weighed technological benefits against potential risks. Although AI was perceived as having the potential to improve efficiency, support generation, and assist analysis, concerns over copyright/IP, academic integrity, algorithmic bias, output reliability, and unclear rules strengthened teachers’ cautious attitudes (S7, S8). Under such risk judgments, teachers were more likely to adopt AI in limited, controlled, or peripheral ways. For example, in the absence of specific guidance for curriculum integration, technology use often remained in low-risk stages such as warm-up, introduction, or assessment, and less frequently entered core activities involving singing, instrumental performance, or music-concept instruction (S2). Therefore, AI adoption was not driven simply by technological availability, but was filtered through teachers’ sense of responsibility, risk judgment, and awareness of professional boundaries.

### 5.3 Triadic moderators and AI adoption strategies

**Figure 3:**
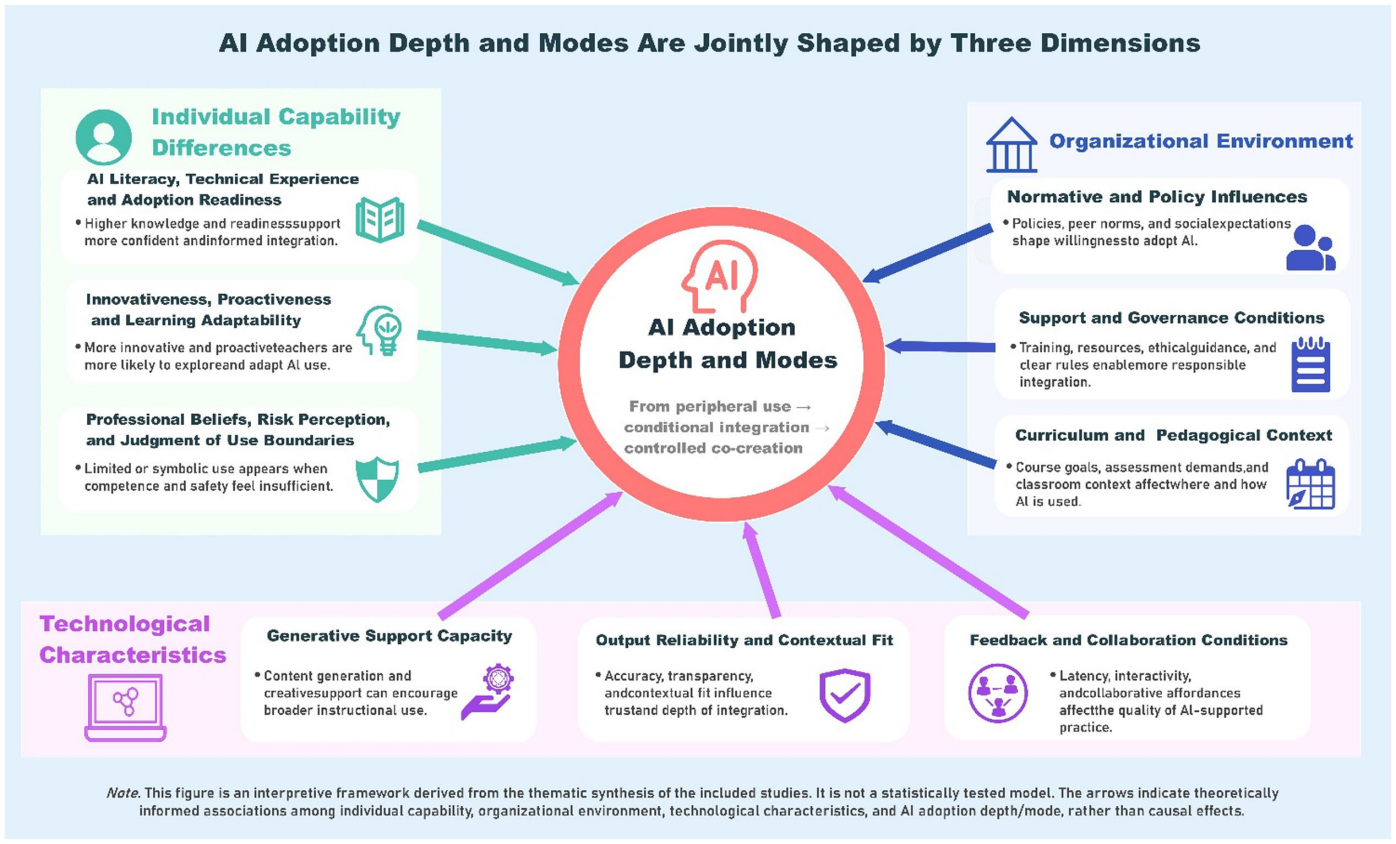
Interpretive framework of triadic moderators and AI adoption strategies derived from thematic synthesis.

Based on the analysis of the included studies, the depth of music teachers’ AI adoption in practice—from peripheral use to more deeply controlled integration—and the mode of adoption—from passive assistance to controlled co-creation—can be understood as a dynamic outcome jointly shaped by individual capability, organizational environment, and technological characteristics (see Figure 3; Table 4).

**Table 4:** Moderating dimensions, core moderators, and AI adoption strategies.

Moderating dimension	Core moderating variable	Specific AI adoption practices and strategies	Study mapping
Individual capability differences	AI literacy, technological experience, and adoption readiness	When teachers have stronger AI knowledge, technological experience, operational confidence, or GenAI-TPACK, they are more likely to move from observational or peripheral use toward prepared curriculum integration, output evaluation, and critical use. When technological knowledge is insufficient, adoption tends to remain in peripheral stages or depend on further training.	S2, S7, S11, S13, S16
	Innovativeness, proactiveness, and learning adaptability	When teachers have higher personal innovativeness, proactive personality, or active learning intention, they are more likely to actively explore AI tools and transform AI from a one-way presentation tool into a support for interactive learning, instructional design, or professional growth.	S5, S18
	Professional beliefs, risk perception, and judgment of use boundaries	When teachers have stronger professional judgment, risk perception, ethical/cultural sensitivity, or creative pedagogical orientation, they are more likely to adopt bounded-use strategies: using AI for creative generation, learning support, or classroom inspiration while retaining human review, critical evaluation, cultural interpretation, and final pedagogical judgment.	S3, S6, S8, S14
Organizational environment	Normative and policy influences	When social influence, education policy, or teacher-education institutional expectations are strong, AI adoption may be driven by external norms. However, if policy is perceived as pressure, it may also inhibit use intention. A more desirable strategy is to translate policy into resources, training, and normative support rather than treating it merely as an external requirement.	S8, S11
	Support and governance conditions	When institutions provide tool access, training resources, ethical guidelines, rule frameworks, privacy protection, and technical support, teachers are more likely to engage in responsible, curriculum-based, and controlled AI integration. When resources, budgets, rules, or ethical governance are lacking, adoption tends to become cautious, constrained, or difficult to sustain.	S7, S9, S17
	Curriculum and pedagogical context	When AI is embedded in specific curriculum goals, teaching tasks, teacher–student relationships, exam-oriented curricula, or teacher-education intervention environments, its use is moderated by classroom structure and pedagogical aims. AI is usually positioned as a support for auxiliary editing, creative inspiration, lesson-plan design, project work, or digital learning resources, rather than as an unbounded substitute for teacher judgment.	S1, S12, S14, S16, S19
Technological characteristics	Generative support capacity	When AI can generate text, images, melodies, harmonies, teaching materials, or creative ideas, teachers are more likely to use it for creative inspiration, material generation, classroom assistance, student project support, and low-threshold creative experience. However, if the generative process induces copying, misinformation, or overreliance, teacher guidance and restrictions are needed.	S4, S7, S9, S14
	Output reliability and contextual fit	When AI outputs show insufficient factual accuracy, hallucinations, contextual mismatch, cultural misinterpretation, privacy/ethical risks, or insufficient algorithmic transparency, teachers are more likely to adopt review-based, revision-oriented, and controlled strategies, using AI to support ideation or feedback but not directly accepting its outputs.	S10, S16, S17, S19
	Feedback and collaboration conditions	When AI or platforms support real-time feedback, multimodal feedback, synchronous/asynchronous collaboration, melody generation, stylistic exploration, or semantic retrieval, AI is more likely to be used for collaborative creation, formative feedback, and online collaborative music learning. However, latency, synchronization problems, audio quality, and feedback overload may limit the depth of use and classroom stability.	S15, S20

First, at the individual level, teachers’ technological cognitive resources and personality traits served as internal drivers of adoption depth. Teachers with systematic GenAI-TPACK competence and higher AI literacy were able to move beyond viewing AI merely as an “efficiency aid,” thereby improving their readiness for integration or supporting lesson-design integration (S7, S16). Meanwhile, teachers with higher personal innovativeness or proactive personality could not only transform AI-induced professional insecurity into active learning intention (S18), but were also more inclined to design highly interactive AI-supported collaborative tasks in the classroom (S5). In addition, teachers with interdisciplinary backgrounds or richer prior experience were better positioned to identify and evaluate AI outputs (S13), and, to some extent, to move beyond traditional barriers of musical skill and engage in exploratory co-creation (S3).

Second, organizational norms and curriculum environments constituted structural boundaries that either constrained or facilitated AI implementation. Clear institutional rules, systematic digital-education training, and sufficient technological resources were important supporting conditions for teachers’ responsible adoption (S8, S9, S12, S17). Conversely, when specific guidance for curriculum integration was lacking, teachers tended to use technology only in peripheral stages such as warm-up, introduction, or assessment (S2). Specific curriculum systems also directly shaped the space for adoption. In strongly exam-oriented curricula, or when constrained by existing curriculum goals and students’ proficiency levels, AI adoption was often pushed into extracurricular experimentation or limited examination-support activities, making it difficult to enter core teaching (S5, S14). Notably, in local contexts with limited resources, low-threshold tools could instead become an important support for teachers to compensate for constraints and promote inclusive education (S6).

Finally, the interactive affordances and performance limitations of AI tools directly shaped the concrete forms of AI intervention in the classroom. Low-threshold generative capacity and multimodal dynamic feedback, such as harmony generation, stylistic exploration, and visualized correction, lowered certain barriers to music participation and enabled teachers to design distributed collaboration and multisensory learning environments (S4, S15, S19). However, the inherent limitations of technology also constrained the breadth and depth of seamless adoption. For example, audio latency and synchronization barriers in online collaborative platforms seriously restricted real-time interaction (S20); the tendency of generative models to hallucinate in specific pedagogical contexts (S16), together with the risks of cultural representational bias and factual inaccuracies associated with non-transparent algorithms (S3, S10, S19), prompted teachers to adopt AI in more cautious and controlled ways. Therefore, due to current technological limitations, AI is mostly restricted to highly structured guidance and supervision, and does not yet support autonomous use without human verification.

Overall, the depth of individual capability shapes the upper limit of exploration, the level of organizational support delineates the boundaries of practice, and the maturity of technological characteristics frames the specific modes of interaction. The coordination and interaction among these three dimensions jointly influence the actual depth and mode of professionals’ AI adoption.

## 6 Discussion and conclusion

### 6.1 Theoretical interpretation of the findings

This study positions music education as a naturalistic observational context for examining professionals’ psychological and cognitive mechanisms, using music teachers as an instrumental case to investigate the psychological responses, cognitive appraisals, and professional decisions they form when engaging with AI tools. Based on a systematic synthesis of 20 included studies, the findings show that music teachers do not simply accept or reject AI. Rather, they continuously negotiate among professional identity, pedagogical responsibility, risk perception, and technological affordances. Specifically, AI adoption was organized around four interrelated psychological–cognitive–professional decision pathways: professional boundaries and retention of judgment authority, learning-process regulation and critical engagement, adaptive co-creation and professional capacity reconstruction, and risk perception with bounded adoption. At the same time, the depth and mode of AI adoption were not determined by technological performance alone, but were jointly shaped by individual capability, organizational environment, and technological characteristics. This pattern is consistent with social cognitive theory, which emphasizes the reciprocal relationship among personal factors, environmental conditions, and behavioral practices (Bandura, 1986, 2001), and also echoes the included studies showing that personal innovativeness, resource support, and technological constraints shape how AI is used in practice (Chatzigiannakis and Papadopoulou, 2025; Li and Wu, 2025; Uzumcu and Acilmis, 2024).

These findings suggest that traditional technology-adoption models need further refinement when AI enters professional educational practice. Classical acceptance models such as UTAUT emphasize the roles of performance expectancy, effort expectancy, social influence, and facilitating conditions in shaping adoption intention (Venkatesh et al., 2003). The present findings partly support this logic, as some studies showed that perceived usefulness, facilitating conditions, education policy, and behavioral intention influenced pre-service music teachers’ or music teachers’ acceptance of AI (He and Ren, 2025; Niu, 2026). However, music teachers did not make adoption decisions simply according to whether AI was “useful” or “easy to use.” Rather, they often recognized AI’s value as a cognitive scaffold for ideation, material generation, preliminary feedback, and information organization, while remaining concerned about its involvement in tasks requiring contextual interpretation, cultural judgment, authorship, or final pedagogical responsibility (Choi, 2023; Dong and Younker, 2025; Jiang, 2025; Li et al., 2025; Weatherly, 2026). Therefore, this pattern further suggests that “paradoxical acceptance” is not merely attitudinal inconsistency, but a selective adoption orientation formed through music teachers’ negotiation among technological convenience, pedagogical value, and professional responsibility.

From the perspective of professional identity, music teachers’ caution toward AI does not necessarily indicate technology resistance. Identity threat theory and self-verification theory suggest that when core professional roles are challenged, individuals tend to preserve a stable sense of professional self-concept, competence, and role legitimacy (Breakwell, 2015; Swann and Read, 1981). In the included studies, such identity-related responses more often appeared as review-based acceptance, bounded delegation, or conditional integration. Teachers retained the right to check, revise, reject, or contextualize AI outputs, especially when AI-generated content conflicted with musical expertise, classroom context, cultural meaning, or pedagogical aims (Cooper, 2026; Dong and Younker, 2025; Li et al., 2025). From this perspective, professional boundary maintenance is not merely defensive conservatism. It may also be understood as a form of psychological regulation and professional responsibility practice: by limiting the scope of AI use, teachers can obtain technological support while preserving perceived control, professional self-efficacy, and judgment responsibility.

The triadic moderator findings further indicate that AI adoption is not the result of a single psychological attitude or a single technological attribute, but a dynamic process shaped by the interaction of individual, organizational, and technological conditions. At the individual level, AI literacy, GenAI-TPACK, prior use experience, personal innovativeness, and proactive personality may increase teachers’ capacity for exploratory AI use (Li et al., 2025; Luo and Liang, 2025; Uzumcu and Acilmis, 2024). At the organizational level, curriculum structures, institutional rules, assessment pressures, training resources, and ethical guidelines may influence whether teachers can transform AI from a peripheral assistive tool into a resource for responsible pedagogical integration (Niu, 2026; Qian, 2023; Shin and Jung, 2024). At the technological level, output reliability, transparency, latency, multimodal affordances, hallucination risk, and cultural bias may determine whether AI is more suitable for controlled co-creation, auxiliary feedback, preliminary generation, or only limited experimentation (Jiang, 2025; Li and Wu, 2025; Qian, 2023).

It should be noted that the overall methodological quality appraisal and evidence-tier classification also call for caution when interpreting these findings. Although the MMAT appraisal of 17 empirical studies indicated an overall moderate-to-high level of methodological quality, the included studies varied considerably in research design, participant type, AI tool, and directness of evidence. In addition, some classroom case studies, philosophical inquiries, and conceptual studies mainly provided auxiliary, contextual, or theory-extending evidence. Therefore, the four pathways identified in this review are better understood as thematic patterns derived from the current evidence base, rather than as psychological mechanisms that have been stably validated across different contexts.

### 6.2 Practical and policy implications

In practical terms, the findings indicate that AI training for music teachers should move beyond tool demonstration or functional introduction and shift toward judgment-oriented professional preparation. First, teachers need task-suitability training to distinguish tasks that are appropriate for AI support, such as ideation, draft generation, preliminary feedback, or material organization, from tasks that require human-led judgment, such as assessment, cultural interpretation, authorship decisions, and final pedagogical decision-making. Second, teachers need AI-output review training, including fact-checking, contextual fit assessment, bias identification, and evaluation of whether AI-generated materials meet disciplinary and pedagogical standards. Third, teachers need to develop a critical-use pedagogy that guides students to compare, question, revise, and justify AI outputs rather than directly accepting generated content. Finally, teachers also need ethical and authorship guidance concerning academic integrity, copyright/IP, data privacy, and responsibility allocation.

At the institutional and policy levels, responsible AI adoption cannot rely solely on individual teachers’ willingness. Without clear rules, stable resources, and technical support, AI use may remain low-risk, peripheral, or symbolic. Conversely, institutions that clarify acceptable and unacceptable AI use, specify when human verification is required, define responsibility for AI-assisted outputs, and provide approved tools and sustained training may reduce teachers’ psychological defensiveness and support cautious but constructive integration. Evaluation criteria for AI-supported teaching should not focus only on efficiency or engagement, but should also include output reliability, disciplinary appropriateness, cultural sensitivity, the degree of teacher oversight, students’ critical engagement, transparency of AI use, and traceability of responsibility. Therefore, governance frameworks should include at least three mechanisms: rules for use boundaries, human-verification procedures, and responsibility-allocation mechanisms. Such institutional arrangements may provide clearer psychological safety boundaries for teachers while protecting professional judgment authority.

### 6.3 Bounded implications for psychology-related professions

Although the evidence comes primarily from music teachers and music education contexts, this review may still provide reference points and implications for psychology-related professions. These implications should not be understood as direct recommendations for clinical or counselling practice. Rather, they should be treated as hypothesis-generating propositions derived from the music-teacher case, which require further examination in real psychological service contexts, including clinical psychology, counselling, psychological assessment, supervision, and occupational support.

First, AI use in psychology-related professions requires stricter task-risk classification. The included evidence indicates that professionals may be more willing to accept AI when it is positioned as a cognitive scaffold, assistive tool, or revisable first-draft generator; however, resistance may increase when AI is perceived as touching final judgment authority or professional responsibility (Dong and Younker, 2025; Jiang, 2025; Li et al., 2025). In psychology-related contexts, this means that AI may be more appropriately limited to low-risk, reviewable, and non-final decision-making tasks, such as information organization, documentation assistance, preliminary resource retrieval, structured note-taking, or non-diagnostic summary generation. By contrast, diagnostic judgment, ethical decision-making, crisis assessment, empathic interpretation, therapeutic relationship management, and responsibility-bearing clinical decisions should remain clearly under human professional authority.

Second, AI-output review in psychology-related professions should not be limited to whether the content is “accurate.” It should also include judgments about client fit, risk level, and responsibility-related consequences. The included studies suggest that the depth of AI integration depends on whether professionals can evaluate output quality, identify bias, understand technological limitations, and maintain ethical awareness (He and Ren, 2025; Niu, 2026). In psychological service contexts, this capacity needs to be further specified as the ability to judge whether AI outputs are appropriate for a particular client, cultural context, risk level, and structure of professional responsibility. Professionals should also document the scope, purpose, review process, and final person responsible for AI use. Therefore, AI literacy in psychology-related professions should not be understood merely as technical operational competence, but should also include risk identification, ethical judgment, and responsibility documentation.

Third, psychology-related institutions that aim to promote responsible AI use need to establish governance and accountability frameworks that are stricter than those typically required in general educational contexts. The included evidence suggests that unclear rules, insufficient resources, weak technical support, or inadequate communication may lead professionals to use AI conservatively, superficially, or only in low-risk peripheral tasks (Qian, 2023; Shin and Jung, 2024). In psychology-related professions, this issue carries greater risk because AI outputs may influence assessment, intervention, documentation, referral, or risk management. Therefore, institutions should clearly define the acceptable scope of AI use, prohibited use cases, human-oversight requirements, data-protection procedures, informed-consent processes, responsibility-allocation mechanisms, and procedures for reviewing and tracing AI-assisted outputs.

Finally, effective AI integration in psychology-related professions is unlikely to emerge naturally from technological performance alone. It is more likely to depend on the three conditions outlined above. However, these conclusions are not direct clinical findings. Rather, they represent evidence-informed directions for future research and should be examined as part of AI research and governance design in psychology-related professions.

### 6.4 Limitations

Several limitations should be considered when interpreting the findings of this review.

First, this review focused on formally published English-language studies from 2023 onwards. This decision helped capture the new phase in which AI entered educational practice after ChatGPT, but it may also have excluded earlier digital music education studies, non-English contexts, and unpublished practice-based reports. As a result, the cultural and institutional diversity of music teachers’ AI-related experiences may not have been fully represented.

Second, the evidence base remains small and emerging. Although 20 studies were included, they varied considerably in research design, participant groups, AI tools, educational contexts, and evidentiary strength. This limits the stability and generalizability of the four pathways. Therefore, these pathways should be understood as exploratory, evidence-informed thematic patterns rather than definitive psychological mechanisms.

Third, music teachers are themselves a highly heterogeneous professional group. The included studies covered general school music education, university contexts, pre-service teacher education, creative disciplines, traditional music, intercultural music, and technology-supported music education. These differences may influence how teachers perceive AI, maintain professional boundaries, and make adoption decisions. Future research needs to further examine whether the pathways identified in this review vary by teaching level, disciplinary specialization, cultural context, professional experience, and prior digital exposure.

Fourth, the included studies did not all provide equally direct evidence of teachers’ psychological processes. Some studies directly examined teacher perceptions, self-efficacy, acceptance, identity, risk perception, or pedagogical judgment, whereas others provided indirect evidence through classroom cases, platform evaluations, system design, or conceptual discussions. Although this review used MMAT quality appraisal and evidence-tier classification to avoid treating all studies as having equal evidentiary weight, some psychological interpretations still relied on theory-informed inference rather than direct measurement in the original studies.

## 7 Conclusion

Overall, this review provides a cautious psychological interpretation of AI adoption in music education. The findings show that music teachers’ AI use is shaped not only by technological affordances, but also by professional identity, cognitive appraisal, pedagogical responsibility, and institutional conditions. Therefore, the main value of this review is not to propose a universal model of AI adoption for all professional groups, but to develop an evidence-informed interpretive framework that can provide theoretical guidance for future research on human–AI collaboration in judgment-intensive professional practice.

## Data Availability

The original contributions presented in the study are included in the article/Supplementary material, further inquiries can be directed to the corresponding author.
