# Supplementary material for: AI-driven psychological and cognitive decision processes in professional practice: a systematic review using music teachers as an instrumental case
Source: Front Psychol. 2026 Jun 15;17:1866711. doi: 10.3389/fpsyg.2026.1866711 (PMC13310918; doi:10.3389/fpsyg.2026.1866711)
Supplement: Supplementary file 1 [file Supplementary_file_1.docx]

Supplementary Material

**Table 1. Database-specific search strategies**

| Database | Search field | Search strategy |
| --- | --- | --- |
| Scopus | TITLE-ABS-KEY | TITLE-ABS-KEY(((music* OR chorus OR choir OR "compose" OR composition* OR conductor* OR band OR "Vocal*" OR composer* OR "choral conduct*" OR solfege OR "ear training" OR singing OR piano OR guitar) AND (educat* OR instruct* OR conservatory OR training OR teach* OR pedagog* OR curricul* OR classroom)) AND (teacher* OR educator* OR tutor* OR facilitator* OR instructor* OR faculty OR professor* OR lecturer* OR "teaching practice*" OR "classroom implementation" OR "teaching role*" OR "pedagogical decision*" OR "pedagogical practice*" OR "pedagogical role*" OR mentor OR coach) AND ("artificial intelligence" OR "AI" OR "generative AI" OR GenAI OR ChatGPT OR "AI-assisted" OR "AI-supported" OR "Intelligent tutoring system*" OR "adaptive system*" OR "automated feedback" OR "recommender system*" OR "music information retrieval" OR "smart classroom*" OR "automated scoring" OR AIGC OR "music generation" OR "automatic assessment" OR "computer-generated" OR Suno OR DeepBach OR MuseNet OR Magenta OR MusicGen OR AIVA OR MusicLM OR Transformer-XL OR "IBM Watson Beat" OR Violy OR Flowkey OR Genially OR Educaplay)) |
| Web of Science Core Collection | Topic / TS | TS=(((music* OR chorus OR choir OR "compose" OR composition* OR conductor* OR band OR "Vocal*" OR composer* OR "choral conduct*" OR solfege OR "ear training" OR singing OR piano OR guitar) AND (educat* OR instruct* OR conservatory OR training OR teach* OR pedagog* OR curricul* OR classroom)) AND (teacher* OR educator* OR tutor* OR facilitator* OR instructor* OR faculty OR professor* OR lecturer* OR "teaching practice*" OR "classroom implementation" OR "teaching role*" OR "pedagogical decision*" OR "pedagogical practice*" OR "pedagogical role*" OR mentor OR coach) AND ("artificial intelligence" OR "AI" OR "generative AI" OR GenAI OR ChatGPT OR "AI-assisted" OR "AI-supported" OR "Intelligent tutoring system*" OR "adaptive system*" OR "automated feedback" OR "recommender system*" OR "music information retrieval" OR "smart classroom*" OR "automated scoring" OR AIGC OR "music generation" OR "automatic assessment" OR "computer-generated" OR Suno OR DeepBach OR MuseNet OR Magenta OR MusicGen OR AIVA OR MusicLM OR Transformer-XL OR "IBM Watson Beat" OR Violy OR Flowkey OR Genially OR Educaplay)) |
| ERIC | / | ((music* OR chorus OR choir OR "compose" OR composition* OR conductor* OR band OR "Vocal*" OR composer* OR "choral conduct*" OR solfege OR "ear training" OR singing OR piano OR guitar) AND (educat* OR instruct* OR conservatory OR training OR teach* OR pedagog* OR curricul* OR classroom)) AND (teacher* OR educator* OR tutor* OR facilitator* OR instructor* OR faculty OR professor* OR lecturer* OR "teaching practice*" OR "classroom implementation" OR "teaching role*" OR "pedagogical decision*" OR "pedagogical practice*" OR "pedagogical role*" OR mentor OR coach) AND ("artificial intelligence" OR "AI" OR "generative AI" OR GenAI OR ChatGPT OR "AI-assisted" OR "AI-supported" OR "Intelligent tutoring system*" OR "adaptive system*" OR "automated feedback" OR "recommender system*" OR "music information retrieval" OR "smart classroom*" OR "automated scoring" OR AIGC OR "music generation" OR "automatic assessment" OR "computer-generated" OR Suno OR DeepBach OR MuseNet OR Magenta OR MusicGen OR AIVA OR MusicLM OR Transformer-XL OR "IBM Watson Beat" OR Violy OR Flowkey OR Genially OR Educaplay) |
| EBSCO (PsycINFO / PsycArticles and RILM) | TI OR AB OR SU | ((music* OR chorus OR choir OR "compose" OR composition* OR conductor* OR band OR "Vocal*" OR composer* OR "choral conduct*" OR solfege OR "ear training" OR singing OR piano OR guitar) AND (educat* OR instruct* OR conservatory OR training OR teach* OR pedagog* OR curricul* OR classroom)) AND (teacher* OR educator* OR tutor* OR facilitator* OR instructor* OR faculty OR professor* OR lecturer* OR "teaching practice*" OR "classroom implementation" OR "teaching role*" OR "pedagogical decision*" OR "pedagogical practice*" OR "pedagogical role*" OR mentor OR coach) AND ("artificial intelligence" OR "AI" OR "generative AI" OR GenAI OR ChatGPT OR "AI-assisted" OR "AI-supported" OR "Intelligent tutoring system*" OR "adaptive system*" OR "automated feedback" OR "recommender system*" OR "music information retrieval" OR "smart classroom*" OR "automated scoring" OR AIGC OR "music generation" OR "automatic assessment" OR "computer-generated" OR Suno OR DeepBach OR MuseNet OR Magenta OR MusicGen OR AIVA OR MusicLM OR Transformer-XL OR "IBM Watson Beat" OR Violy OR Flowkey OR Genially OR Educaplay) |

**Table 2. Characteristics of included studies**

| No. | Citation | Research Method | Research Design | Country | Participants | AI Tool / Technological Context | Key Findings |
| --- | --- | --- | --- | --- | --- | --- | --- |
| S1 | Dong, X., & Younker, B. A. (2025). A philosophical inquiry into utilizing ChatGPT through an I-Thou framework. Action, Criticism, and Theory for Music Education, 24(3), 109–137. | Qualitative | Philosophical inquiry | Canada | Reflective interaction between one doctoral student and supervisor | ChatGPT-4 for manuscript editing and proofreading | ChatGPT-4 can undertake initial editing and proofreading, but the supervisor/human expert still needs to retain critical review, meaning-making, and control over authorial voice. |
| S2 | Shin, J., & Jung, J. Y. (2024). An investigation of Korean music teachers’ perceptions of technology in music classes. International Journal of Music Education. Advance online publication. | Mixed Methods | Survey and semi-structured interviews | South Korea | Korean music teachers | General music technology, AI, metaverse, sequencing, and related tools | Korean music teachers recognized the potential pedagogical value of technology, but its use was mainly concentrated in warm-up, opening, or assessment stages; use in core singing, instrumental, and music-concept activities remained limited. |
| S3 | Jiang, R. (2025). Breaking the boundaries: Philosophical encounters with artificial intelligence in music education. Action, Criticism, and Theory for Music Education, 24(3), 53–78. | Qualitative | Philosophical inquiry | Canada | Two experiential examples involving the author and Q | Generative AI music platforms, Mubert, and a self-built AI model | Generative AI can lower barriers to music creation and expand participation for those excluded by traditional music education, but bias, database transparency, cultural misinterpretation, and human musical agency need to be critically addressed. |
| S4 | Choi, M. (2023). Design, implementation, and effects of elementary music creation class using an AI-based music program, Doodle Bach. Korean Journal of Research in Music Education, 52(4), 211–237. | Mixed Methods | Teaching experiment with interviews and survey | South Korea | Elementary students and teachers | Doodle Bach; AI-assisted harmony/composition program | Doodle Bach can serve as an auxiliary tool in elementary music creation by offering help and inspiration, but the timing of AI use, teacher guidance, and the design of students’ critical thinking remain crucial. |
| S5 | Uzumcu, O., & Acilmis, H. (2024). Do innovative teachers use AI-powered tools more interactively? A study in the context of diffusion of innovation theory. Technology, Knowledge and Learning, 29, 1109–1128. | Mixed Methods | Case study | Türkiye | 32 pre-service teachers | AI-powered educational tools | Pre-service teachers’ personal innovativeness was related to whether they designed student–AI tool interaction in lesson plans; more innovative participants were more likely to adopt interactive AI teaching designs. |
| S6 | Chatzigiannakis, D., & Papadopoulou, A. (2025). Educational equity at the geopolitical frontier: A music-based teacher training case. IAFOR Journal of Education: Studies in Education, 13(3), 643–672. | Qualitative | Case study | Greece | 36 primary and secondary music teachers | AI-assisted/open-source tools; music creation, sound editing, and AI-generated soundtracks | Short-term music teacher training enhanced teachers’ creative self-efficacy, inclusive pedagogical awareness, and contextualized pedagogical imagination when using low-threshold AI/open-source tools. |
| S7 | Basty, R., Kropczynski, J., & Halse, S. (2025). Empowering faculty in creative and cultural disciplines: AI literacy and image generator integration in higher education. Journal of Information Technology Education: Innovations in Practice, 24, Article 27. | Mixed Methods | pre-post test + focus group | United States | 51 faculty members in creative and cultural disciplines | AI image generators | The AI literacy workshop improved faculty confidence, understanding, ethical awareness, and readiness to integrate image generators into curricula; however, tool access, budget constraints, academic integrity, accountability, and copyright/IP risks limited responsible adoption. |
| S8 | He, S., & Ren, Y. (2025). Exploring pre-service music teachers’ acceptance of generative artificial intelligence: A PLS-SEM-ANN approach. Frontiers in Psychology, 16, Article 1571279. | Quantitative | Survey study | China | Pre-service music teachers | Generative AI in pre-service music teacher education | Among pre-service music teachers, behavioral intention, perceived risk, and facilitating conditions were important predictors of actual GenAI use; the study mainly provided technology-acceptance model evidence rather than evidence of actual classroom professional decision-making. |
| S9 | Kladaki, M., Kostas, A., & Alexopoulos, P. (2025). Exploring teachers’ beliefs about ChatGPT in arts education. Education Sciences, 15(7), Article 795. | Qualitative | Semi-structured interviews | Greece | 67 arts teachers familiar with or using ChatGPT | ChatGPT in arts education | Arts teachers saw the value of ChatGPT for research, material generation, and pedagogical support, but were also concerned about copying, misinformation, reduced critical thinking, and declining creativity; they emphasized the importance of training, rule frameworks, institutional support, and technological infrastructure. |
| S10 | Parmar, D., Vignesh, R., Faiz, A., Narayana, A., Hembade, S. C., & Sharma, P. (2025). Integrating ChatGPT for music history education. ShodhKosh: Journal of Visual and Performing Arts, 6(1s), 468–477. | Mixed Methods | Controlled Experimental Design + interview | India | Mainly students; teacher-related evidence was indirect | ChatGPT for music history education | ChatGPT can function as a cognitive scaffold and virtual assistant in music history learning, but factual accuracy, content consistency, and overreliance indicate the need for structured guidance and human judgment. |
| S11 | Niu, Y. (2026). Modeling music student teachers’ behavioral intention of using artificial intelligence in China. Frontiers in Psychology, 17, Article 1756135. | Quantitative | Survey study | China | Music student teachers | AI use in music teacher education | Pre-service music teachers’ intention to use AI was jointly influenced by performance expectancy, effort expectancy, social influence, facilitating conditions, and education policy; education policy could operate as direct pressure and indirectly support adoption through social influence and effort expectancy. |
| S12 | Aliksiichuk, O., Borysova, T., Kartashova, Z., Priadko, O., Kuziv, M., & Chaban-Chaika, S. (2025). Modern digital approaches to training music teachers: Evolution from classical to interactive. International Journal on Culture, History, and Religion, 7(Special Issue 1), 273–296. | Qualitative | Qualitative descriptive design | Ukraine | Future music teacher training context | AI tools, digital platforms, specialized software, and simulators | Digital/AI tools can supplement classical music teacher training and support ear training, rhythm, music reading, audio processing, and methodological training, but the authors emphasized that digital technologies cannot replace classical training and live teacher–student interaction. |
| S13 | Cooper, P. K. (2026). Music teachers’ labeling accuracy and quality ratings of lesson plans by artificial intelligence (AI) and humans. International Journal of Music Education, 44(1), 23–36. | Mixed Methods | Survey study + Qualitative open-ended responses | United States | Music teacher raters | AI-generated lesson plans / GPT-generated lesson plans | Music teachers had difficulty distinguishing AI-generated lesson plans from human-written ones; prior AI-use experience was related to identification accuracy, and teachers’ judgments mainly relied on specific details, classroom knowledge, and wording cues. |
| S14 | Weatherly, K. I. C. H. (2026). Navigating the intersection of generative artificial intelligence and democratic pedagogy in music education in Macau. British Journal of Music Education. Advance online publication. | Qualitative | Case study | China | One music teacher, Adam; one-year case | GenAI tools, Soundtrap, and songwriting AI | GenAI can support lesson planning, sound experimentation, creative inspiration, and differentiated support, but its deterministic and generic outputs may constrain students’ deeper creative agency, original thinking, and critical engagement. |
| S15 | Jiang, H. (2026). Posthumanism in cultivating children’s musical creative practice: A theoretical framework and case study from a primary school. Music Education Research, 28(1), 66–79. | Qualitative | Case study | China | Children’s classroom case; teacher evidence was weak | AI music tools with natural materials and ambient soundscapes | In the primary-school case, students used AI music tools as collaborators for lyric writing, melody generation, and stylistic exploration; the study mainly provided evidence for posthumanist educational design and classroom cases, while teacher psychological/decision evidence was weak. |
| S16 | Li, H., Chai, C. S., & Wang, X. (2025). Promoting pre-service music teachers’ TPACK with generative AI: An intervention through designing with AI. Computers and Education: Artificial Intelligence, 9, Article 100525. | Mixed Methods | Single-group pre-post test + Semi-structured interviews | China | 59 pre-service music teachers | GenAI-assisted lesson design within the 5E instructional model | The design-based GenAI intervention significantly improved pre-service music teachers’ GenAI-specific TPACK, self-efficacy, and critical understanding, and supported their use of GenAI for ideating, evaluating, and revising lesson plans while retaining professional judgment. |
| S17 | Qian, C. (2023). Research on human-centered design in college music education to improve student experience of artificial intelligence-based information systems. Journal of Information Systems Engineering and Management, 8(3), Article 23761. | Qualitative | Semi-structured interview study | China | Mixed teacher and student evidence | AI-based information systems in college music education | AI-based information systems can support data analysis, personalized feedback, learning support, and collaborative projects; effective integration requires human-centered design, teacher training, curriculum alignment, technical support, privacy protection, and ethical frameworks. |
| S18 | Luo, M., & Liang, S. (2025). Resistance or resilience? University music teachers’ active learning intention in response to the emergence of artificial intelligence. Acta Psychologica, 261, Article 105775. | Quantitative | Survey study | China | University music teachers | AI as a perceived technological impact in university music teaching | University music teachers’ technology impact awareness directly promoted active learning intention; proactive personality strengthened the transformation from job insecurity to active learning intention, but the study mainly measured learning intention rather than actual classroom adoption behavior. |
| S19 | Li, N., & Wu, D. (2025). The auxiliary function and realization mechanism of artificial intelligence in cross-cultural traditional music education. Journal of Cases on Information Technology, 27(1), 1–18. | Quantitative | Randomized Controlled Trial | China | Learner/system application-focused; teacher evidence was indirect | Multimodal semantic mapping, dynamic demonstration, and interpretable panel | AI can reduce rhythm errors, support dynamic demonstration, and provide multimodal feedback in cross-cultural traditional music education; however, emotional-symbol recognition, feedback latency, cultural-bias correction, and algorithmic transparency limit its higher-order cultural interpretation functions. |
| S20 | Alexandraki, C., & Tsioutas, K. (2025). Web-based collaborative music lessons: Approaches, challenges and perspectives. Journal of the Audio Engineering Society, 73(12), 849–861. | Mixed Methods | Design-Based Research | Greece | Teacher/instructor needs survey and platform evaluation | MusiCoLab, audio-to-score alignment, semantic interactions, and latency compensation | MusiCoLab supports synchronous/asynchronous online collaborative music learning and semantic interaction, but latency, synchronization, audio quality, and user-experience issues constrain the quality and scalability of collaborative music teaching. |

**Table 3. Comprehensive Coding Matrix and Evidence-to-Theme Mapping of Included Studies**

| No. | Original Evidence | Professional Response / Decision | Evidence for Professional Response | Primary Pathway | Secondary Pathway | Additional Secondary Pathway | Individual Capability Differences | Evidence: Individual Capability Differences | Organizational Environment | Evidence: Organizational Environment | Technological Characteristics | Evidence: Technological Characteristics | Influence on AI Adoption Depth and Mode | Evidence: AI Adoption Depth and Mode |
| --- | --- | --- | --- | --- | --- | --- | --- | --- | --- | --- | --- | --- | --- | --- |
| S1 | “My supervisor reviews the AI-edited text, providing insight and critical feedback.” (procedural reflection) | ChatGPT was used for first-round editing and proofreading, while the supervisor retained review, feedback, and meaning judgment. | “I then utilize ChatGPT-4 for the first-round editing and proofreading. My supervisor reviews the AI-edited text, providing insight and critical feedback.” (procedural reflection) / “the supervisor experienced a shift from the dominant teacher to a ‘scrutinizer,’ carefully examining ChatGPT’s contributions...” (conclusion) | Path 1 | Path 3 |  | Supervisor acceptance and the student’s reflective AI-use competence | “The student’s clarity about the value of ChatGPT and how she utilized it contributed to my acceptance of its use.” (supervisor reflection) | Supervisor–student authority relations and academic supervision structure | “When the third party, in this case ChatGPT, joins the traditional supervision process, the power dynamics starts to transform to a more balanced status.” (supervisee reflection) | Generative editing capacity and limitations in prompt adherence | “Although there were instances of ChatGPT deviating from my prompts, it did not require much time or effort to bring it back on course.” (supervisee reflection) | AI undertook initial editing/auxiliary feedback, while humans retained review, meaning judgment, and control over authorial voice. | “the supervisor experienced a shift from the dominant teacher to a ‘scrutinizer,’ carefully examining ChatGPT’s contributions and offering insights to improve the AI’s effectiveness.” (conclusion) |
| S2 | “infrequently used music technology in main activities such as teaching musical concepts, musical modeling/practice, and review.” (author summary of survey results) | Technology was mostly used in warm-up, opening, or assessment stages, and less often in core music activities. | “they tend to use technology either in the warm-up/opening activity or assessment parts...” (survey results) / “infrequently used music technology in main activities...” (author summary of survey results) | Path 4 |  |  | Limited knowledge and experience with smart technologies | “Korean music teachers have limited knowledge of fourth industrial revolution smart technologies, they believe AI and the metaverse will be helpful for teaching music.” (discussion) | Need for teacher education and professional development support | “music teachers need to be given opportunities to participate in teacher education programs and professional development workshops that give them fundamental knowledge about AI and its possible applications in music education.” (discussion) | Potential for curriculum expansion alongside limited use in core activities | “more than half of participants responded that they tend to use technology either in the warm-up/opening activity or assessment parts of their music classes, reporting that they infrequently used music technology in main activities such as teaching musical concepts, musical modeling/practice, and review.”(Result); “music teachers place on the abilities of music technologies to give their students access to otherwise inaccessible information and resources.” (discussion) | When technology is introduced only at the functional level, adoption tends to remain peripheral; with specific curriculum-integration guidance, the scope of adoption may expand. | “In addition, 29.5% of participants reported that they rarely use music technology for singing, while 24.8% indicated that they do not incorporate technology for playing instruments… indicating teachers tend to rarely use technology for performing activities.”(Result);“Encouraging music teachers to embrace the full potential of various technologies will require explaining how these technologies can be integrated into a broad music curricula, instead of simply introducing and explaining their elements and functions.” (discussion) |
| S3 | “All the music AI models are biased because those who build them are AI scientists and they must have biases.” (case evidence/direct statement); “Through one or multiple hidden layers of neuron connections run by algorithms, AI goes through intricate learning procedures not fully disclosed to humans...”(discussion) | AI can be used to expand musical participation and co-creation, but data, cultural representation, transparency, and ethical issues require critical handling. | “Q insisted on building a music AI model on their own rather than using one.” (case evidence) / “Relying on commercial AI software... may misinterpret music genres and cultures...” (discussion) / “developing and using AI in more interactive, transparent, and ethical ways.” (conclusion; author implication) | Path 3 | Path 1 |  | Technical expertise and cross-domain music-creation capacity | Q’s purpose in building this AI model was to explore alternative ways of engaging with music composition that align with their personal interests and background in information technology(Case) | Knowledge barriers and participation structures in traditional music education | “traditional performance-oriented approaches often enable socioeconomically and educationally privileged groups to access music activities that are compatible with their social backgrounds” (author discussion) | Low-threshold generation and co-creative capacity, alongside risks related to data/database transparency | “without learning to write R&B drum patterns or using composition software on my computer, I became a co-creator of an R&B song excerpt using AI within a minute.” ; “Generative AI, by breaking the boundaries between users’ aspirations and their actual capabilities, becomes a transformative tool for music learning and making.”(Empirical Practices with Music AI) ; “Relying on commercial AI software for music creation may misinterpret music genres and cultures due to its non-transparent music database, unobservable learning process, and hallucinations and misinterpretations of music genres.”(Discussion) | Reflective and critical adoption: AI is used to expand participation and co-creation while retaining critical attention to culture, transparency, and ethics. | “AI technology acted as an empowering tool for both Q and myself, enabling us to engage with music learning and creation in ways that we could not achieve based on our backgrounds.” (A Sociological Lens and Musician’s Agency);“Q insisted on building a music AI model on their own rather than using one.” (discussion) “All the music AI models are biased because those who build them are AI scientists and they must have biases.”(A Sociological Lens and Musician’s Agency) |
| S4 | “it was suggested that the design of the lesson should allow students to think critically.” (Teacher A interview excerpt) | Teachers controlled the timing of AI intervention and used it as a creative support tool under teacher guidance. | “The role of the teacher and the timing of AI tool usage were deemed crucial.” (Teacher A interview excerpt) / “there is a need to refine the timing of introducing AI as a tool in the classroom and to emphasize the importance of the teacher's role...” (discussion/conclusion) | Path 2 | Path 3 |  | Teachers’ judgment about learning processes and pedagogical regulation | “it was suggested that the design of the lesson should allow students to think critically.” (Teacher A interview excerpt) | Peer feedback, sharing of creative works, and classroom composition-process design | “both teachers agreed that the process of sharing creative works and providing feedback among peers, which was integrated into the course, proved to be highly effective in fostering students' creative processes and learning.” (author summary of teacher interviews) | AI harmony generation, inspiration support, and low-threshold musical experience | “artificial intelligence can serve as an assistant tool in the music composition ‘process,’ enabling students to have musical experiences easily, even if they lack specialized musical knowledge or skills” (author analysis) | Guided/controlled classroom adoption: AI was introduced as an auxiliary tool, while teachers controlled the timing of use and the learning process. | “there is a need to refine the timing of introducing AI as a tool in the classroom and to emphasize the importance of the teacher's role during the entire process.” (discussion/conclusion) |
| S5 | None | Pre-service teachers with higher innovativeness were more likely to design student–AI tool interaction in lesson plans. | “innovative preservice teachers could provide more student interaction with AI-powered tools.” (discussion; lesson-plan analysis interpretation) | Path 3 |  |  | Personal innovativeness | “As the interaction level increased, so did the innovativeness mean ranks.” (quantitative result) | Curriculum fit, student level, and classroom constraints | “The participants especially had difficulty adapting the tools to the primary school music curriculum achievements.” (author summary of content analysis) | Personalized interaction, feedback, and gamified features | “One of the main features of AI-powered tools is that they promote an individualized learning environment through interaction.” (discussion) | Interactive adoption increased with personal innovativeness, although future use intention remained limited. | “It showed that innovative preservice teachers could provide more student interaction with AI-powered tools.” (discussion) |
| S6 | “A key finding was the significant boost in participants’ creative self-efficacy during and after their engagement with the tools introduced.” (author findings) | Teachers connected sound, AI/open-source tools, local classroom needs, inclusive goals, and students’ expressive opportunities. | “Teachers moved from tentative experimentation to context-specific imagining...” (thematic analysis/author synthesis) / “the musical and digital tools served as catalysts for teacher expression and inclusive design.” (thematic analysis/author synthesis) | Path 3 | Path 4 |  | Creative self-efficacy and willingness to experiment | “A key finding was the significant boost in participants’ creative self-efficacy during and after their engagement with the tools introduced.” (author findings) | Insufficient systemic support and local resource constraints | “When the system doesn’t support us, at least we can build something of our own. Sound helps me give shape to my class”. (participant reflection) | Low-threshold tools and support for creative agency | “The workshop revealed that reclaiming creative agency does not require advanced technical skills or institutional reform; it begins with low-barrier tools and imaginative practice.” (discussion) | Training helped teachers connect AI/open-source tools with local classroom needs, inclusive goals, and opportunities for student expression. | “Teachers moved from tentative experimentation to context-specific imagining, underscoring how arts-integrated technologies can stimulate professional insight.” ; “the musical and digital tools served as catalysts for teacher expression and inclusive design.” ; “The codes reflect a dual movement: inward, toward renewed confidence, and outward, toward shared educational commitment.” (thematic analysis/author synthesis) |
| S7 | “faculty showed a positive attitudinal shift. They reported increased confidence, pedagogical and ethical awareness...” (Findings) | Faculty prepared to integrate image generators through AI literacy training, ethical reflection, and curriculum design. | “Post-workshop feedback indicated... confidence, understanding, and readiness to integrate image generators...” (discussion; post-workshop feedback) / “They expressed appreciation for image generators as complementary tools and readiness to integrate them into their teaching.” (findings) | Path 4 | Path 3 | Path 1 | Improved AI literacy, understanding, confidence, and integration readiness | “faculty showed a positive attitudinal shift. They reported increased confidence, pedagogical and ethical awareness, access to institutional resources, and practical applications post-workshop.”(Findings)/ “Post-workshop feedback indicated that the designed workshop positively impacted faculty’s perceived confidence, understanding, and readiness to integrate image generators into their creative and cultural courses.”(Discussion) | Tool access, resource constraints, and need for ethical guidelines | “institutional barriers that challenged faculty in adopting image generators into their department included limited access to tools, as well as resources and budget constraints.”(post-workshop survey summary)/ “Institutions should support faculty development through accessible AI tools, discipline-based literacy resources, hands-on training, and ongoing learning opportunities tailored to them. Interdisciplinary collaboration and developing ethical guidelines are essential for responsible GenAI adoption.”(Recommendations for Practitioners) | Image generators as complementary teaching tools, with academic integrity, accountability, and copyright/IP risks | “They expressed appreciation for image generators as complementary tools and readiness to integrate them into their teaching.”(Findings)/ “Academic Integrity,” “Accountability,” and “Copyright & IP” were the top three consistently ranked with higher risk severity by multiple participants.”(poll summary) | With structured AI literacy workshops, accessible tools, resource support, and ethical guidelines, teachers were more likely to develop confidence, understanding, and readiness to integrate image generators into courses; limited access, budgets, and ethical/IP risks constrained responsible adoption. | “Post-workshop feedback indicated that the designed workshop positively impacted faculty’s perceived confidence, understanding, and readiness to integrate image generators into their creative and cultural courses.”(Discussion)/ “institutional barriers... included limited access to tools, as well as resources and budget constraints.”(post-workshop survey summary)/ “developing ethical guidelines are essential for responsible GenAI adoption.”(Recommendations for Practitioners) |
| S8 | “Perceived Risk (PR, 74.11%) and Facilitating Conditions (FC, 48.14%) were the next most important factors.” (ANN sensitivity analysis) | The article mainly provided an acceptance model and recommendations for updating teacher education curricula. | “can guide higher education institutions in updating their training programs...” (practical implications; author recommendation) | Path 4 |  |  | Behavioral intention, perceived risk, facilitating conditions, and lower-importance habit and compatibility | “As shown in Table 7, in Model B, the most prominent predictor of Actual Usage Behavior (UB) was Behavioral Intention (BI), with a normalized importance of 100%, indicating that it was the most critical factor influencing UB. Following BI, Perceived Risk (PR, 74.11%) and Facilitating Conditions (FC, 48.14%) were the next most important factors.” (ANN sensitivity analysis) | Teacher training and institutional curriculum updating | “The feedback from pre-service music teachers on their acceptance of generative AI can guide higher education institutions in updating their training programs and designing more effective, targeted courses.” (practical implications) | Compatibility between generative AI and existing teaching models | “The data indicate that the pre-service music teachers surveyed believe that generative AI technology can be compatible with current teaching practices and has the potential to enhance the teaching model.” (discussion) | Actual use behavior was mainly influenced by behavioral intention, perceived risk, and facilitating conditions. | “As shown in Table 7, in Model B, the most prominent predictor of Actual Usage Behavior (UB) was Behavioral Intention (BI), with a normalized importance of 100%, indicating that it was the most critical factor influencing UB. Following BI, Perceived Risk (PR, 74.11%) and Facilitating Conditions (FC, 48.14%) were the next most important factors.”; “The feedback from pre-service music teachers on their acceptance of generative AI can guide higher education institutions in updating their training programs and designing more effective, targeted courses. This can help alleviate some of the resistance and misconceptions pre-service music teachers may have toward technology...”(Practical implications) |
| S9 | “the lack of knowledge and experience in using ChatGPT in the arts was identified as a key barrier...” (Results—Control beliefs) | Teachers believed that clear rule frameworks, institutional support, and curriculum-integration conditions were needed before using ChatGPT. | “the helpful role of institutional support was pointed out, meaning the presence of a clear framework of rules...” (Results—Control beliefs/author summary) | Path 2 | Path 4 | Path 1 | Personal confidence, knowledge/experience, and perceived behavioral control | “the personal confidence of each teacher that they will be able to use ChatGPT in arts lessons was also identified and recorded as a helpful factor (n = 35, n% = 9.54%, Participant 24: ‘I also feel quite a lot of personal confidence that I can use it myself’).” (interview thematic results) | Institutional support and rule frameworks | “the helpful role of institutional support was pointed out, meaning the presence of a clear framework of rules for the use and proper pedagogical exploitation of ChatGPT by students and also by teachers” (author summary) | Rapid responses, research support, and assistant functions, alongside risks of misinformation and copying | “Furthermore, the contribution of ChatGPT to research was also noted due to the speed of responses (n = 43, n% = 12.11%, Participant 26: ‘They would have answers for every question they had. For example, they could ask about a painting or an artwork’).” (Results—Behavioral beliefs) / “The teachers also highlighted its potential contribution as an assistant (n = 33, n% = 9.30%) in a variety of ways, such as providing materials, ideas, and exercises, saving time, and creating teaching scenarios.” (Results—Behavioral beliefs) / “Among the concerns, the most frequent was the mention of the potential risk of students using ChatGPT to copy and avoid personal effort...” (Results—Behavioral beliefs) / “ChatGPT may contribute to the inactivation of thinking and the reduction in students’ critical faculties...” (Results—Behavioral beliefs) / “several concerns... were also expressed about the misinformation it may provide...” (Results—Behavioral beliefs) | Teachers perceived both pedagogical value and risks; when teachers lacked knowledge/experience, faced curriculum-integration difficulties, or lacked rule frameworks, classroom use was more constrained. | “In particular, the lack of knowledge and experience in using ChatGPT in the arts was identified as a key barrier (n = 83, n% = 22.62%).” (Results—Control beliefs) / “In addition, teachers reported the difficulty of integrating it into the existing educational system and curriculum as a barrier...” (Results—Control beliefs) / “the helpful role of institutional support was pointed out, meaning the presence of a clear framework of rules for the use and proper pedagogical exploitation of ChatGPT by students and also by teachers” (Results—Control beliefs / author summary) |
| S10 | “AI should not substitute human judgment, but support it...” (discussion) | ChatGPT was used in guided learning contexts with structured guidance and fact-checking. | “guided learning contexts...” (author interpretation) / “Reliability... concerns of the accuracy of facts and the consistency of the content.” (case-study analysis) |  | Path 1 | Path 2 | None | None | Guided course structure and teacher supervision | “ChatGPT could successfully be applied in guided learning contexts to improve both academic performance and interest in the study of music history.” (author interpretation) | Conversational cognitive scaffolding and contextual support, but weaker factual accuracy and content consistency | “ChatGPT contributed to understanding significantly, as it gave the students an opportunity to debate the complicated history events, asking questions and giving them feedback of contents.”(Discussion); “Reliability, however, (mean = 4.0) was rated relatively lower that raises the concerns of the accuracy of facts and the consistency of the content” (case-study analysis) | In guided learning contexts, ChatGPT can serve as a cognitive scaffold and virtual assistant; however, factual accuracy and content consistency require structured guidance during use. | “ChatGPT was also successfully employed as a cognitive scaffold and virtual co-teacher in the collaborative conversation, thoughtful processing, and exploratory contextualization of musical periods.” (conclusion) |
| S11 | “policy-related expectations may be perceived... as external pressure...” / “education policy functions as structural support...” (discussion) | Adoption intention was stronger when AI was perceived as useful, easy to use, socially accepted, and appropriately supported by policy; excessive policy pressure might suppress intention. | “Performance expectancy, effort expectancy and social influence exerted significant and positive influences...” (discussion) / “education policy produced... negative direct impact... strong indirect effect...” (indirect-effect analysis) | Path 4 |  |  | Performance expectancy, effort expectancy, AI-use habit, and behavioral intention | “performance expectancy (β = 0.481, p < 0.001), effort expectancy (β = 0.333, p < 0.001), social influence (β = 0.811, p < 0.001), facilitating conditions (β = −0.143, p < 0.05), and education policy (β = −0.607, p < 0.001) all exerted statistically significant influences.” (hypotheses testing) | Social influence, education policy, and facilitating conditions | “music student teachers’ decisions about AI in future practices are highly influenced by their mentors, peers and institutional expectations” (discussion) | Perceived usefulness, perceived ease of use, and complexity of professional tools | “performance expectancy (β = 0.481, p < 0.001), effort expectancy (β = 0.333, p < 0.001), social influence (β = 0.811, p < 0.001), facilitating conditions (β = −0.143, p < 0.05), and education policy (β = −0.607, p < 0.001) all exerted statistically significant influences.”(quantitative result); “As AI music tools often involve complex interfaces such as AI scoring systems and performance recognition algorithms, teacher education providers should reduce the cognitive load of learning and increase hands-on demonstrations and practices with music-focused technologies.” (discussion); “The proposed model explained 62.4% of the variance in music student teachers’ willingness and there are five variables, performance expectancy, effort expectancy, social influence, facilitating conditions, and education policy, emerging as significant determinants.”(discussion summary of model results) | Policy had both direct and indirect effects: it could suppress use intention as external pressure, but also indirectly promote adoption through social influence, effort expectancy, resources, and training. | “At the direct level, policy-related expectations may be perceived by student teachers as external pressure and potentially elicit their psychological resistance. At the indirect level, education policy functions as structural support that shapes normative expectations and allocates resources and training.” (discussion) |
| S12 | “digital platforms allow achieving a number of strategic goals of professional training of music teachers...”(Results); “there are psychological limitations in digital learning... weakening of personal contact between student and teacher...” (Discussion; author synthesis) | The authors recommended integrating digital/AI tools into teacher-training curricula for ear training, rhythm, music reading, audio processing, and methodological training. | “can be used to develop new teacher training programs... specialized digital simulators...” (Discussion; author synthesis) | Path 4 | Path 1 |  | Insufficient digital literacy and variation in teacher readiness | “the barriers include the lack of readiness of individual teachers to organize learning in a digital environment, which requires systematic training of teaching staff in digital pedagogy” (Results/author synthesis) | Platform resources, digital divide, and systematic training support | digital platforms allow achieving a number of strategic goals of professional training of “music teachers, in particular: providing open access to educational material; forming an individualized educational trajectory; creating an interactive environment for the implementation of music-theoretical, performance and methodological tasks; providing constant pedagogical support for students; stimulating the reflective and creative activity of future teachers; developing self-learning and self-organization skills...”(author synthesis); “Despite the obvious advantages of using digital platforms, it is also worth considering the challenges associated with their implementation. In particular, the barriers include the lack of readiness of individual teachers to organize learning in a digital environment, which requires systematic training of teaching staff in digital pedagogy. Another problem is the digital divide, i.e., teacher education students' unequal access to digital resources.”(author synthesis); “Among them, authors have identified: the digital divide, the insufficient level of digital literacy of teachers, and the lack of systematized methodological resources for the effective implementation of digital learning in the field of art education.”(Discussion) | Interactive platforms, music-text recognition, and audio/rhythm analysis capabilities | “digital platforms... including: forums for professional communication, blogs for recording pedagogical reflections, e-mail for counseling, chats for synchronous discussion... and wikis for the collective development of methodological projects” (platform-function analysis / author synthesis) / “creating digital scores, editing music notation, exporting material in PDF and MP3 formats, and changing instrumental sound...” (specialized software analysis / author synthesis) / “Pedagogical simulators that integrate interactive tools...” (specialized software analysis / author synthesis) / “machine learning algorithms for analyzing rhythmic structures, classifying musical fragments, and processing music notation...” (Conclusions / author synthesis) | With digital platform resources, systematic training, and methodological resources, teacher training can shift from reproductive learning toward productive and blended digital learning. | “transitioning from a reproductive to a productive learning model through digital technologies...” (author synthesis) / “Future music teachers can create their own musical content: phonograms, scores, and interactive lessons.” (author synthesis) |
| S13 | “Overall, music teachers did not find the task to be easy and lacked confidence in their ratings.” / “specific details, evidence of classroom knowledge, and wording.” (Results) | No direct classroom adoption decision evidence | None | Path 1 |  |  | Prior AI-use experience and ability to identify lesson-plan source | “Personal use of AI .139 .362 .018” (Table 4; regression model) ; “Labeling accuracy was positively predicted by quality scores on human-made lesson plans and previous personal use of AI...” (Abstract / Results summary) | None | None | Confusability and quality differences of AI-generated lesson plans | “Music teachers could not accurately label if a lesson plan was created by humans or generated by AI despite AI lesson plans being rated lower in quality than human-made lesson plans.” (Discussion) | Teachers relied mainly on specific details, classroom knowledge, and wording cues when judging AI-generated lesson plans, suggesting that if AI lesson plans enter actual teaching, they still require teacher quality review based on classroom experience and professional knowledge. | “Three overarching themes emerged... specific details, evidence of classroom knowledge, and wording.” /(Results); “Overall, music teachers did not find the task to be easy and lacked confidence in their ratings.” (Results); “The open-ended responses would indicate assessors were most confident a lesson plan was made by AI when they noticed a lack of specificity or if the musical or pedagogical knowledge didn’t meet the needs they expected in their own classrooms.” ( Discussion) |
| S14 | “my concern arises when there is no trace of original thought—just generic, AI-generated content.” (teacher interview) | The teacher shifted the focus from mastering AI tools to using AI to inspire creativity, confidence, and experimentation, while guiding students to use AI critically. | “The focus is no longer on mastering the AI tool itself but on how these tools can inspire creativity, confidence, and experimentation...” (Findings; teacher case/author report) / “my concern arises when there is no trace of original thought—just generic, AI-generated content.” (teacher interview) | Path 2 | Path 3 | Path 1 | Teacher’s creative pedagogical orientation and critical guidance tendency | “Rather than focusing on technical mastery of music production software, Adam emphasised creativity, exploration and practical application.”(author report) / “I encouraged them to engage with AI in a more dialogic manner, critically assessing the answers provided...” (Adam interview)/ “he worried that the overuse of AI-generated music could hinder the development of a student’s individual creative voice.”(Findings) | Exam-oriented curriculum and limited integration space | “limited space for integration of GenAI within an examination-oriented curriculum” (Findings) | AI-assisted composition, melody generation, and cross-style sound experimentation | “he guided students in using Soundtrap, an AI-powered digital audio workstation (DAW), to compose original pieces, encouraging them to experiment with different genres and sounds without fear of making mistakes.”(Findings); “they utilised a songwriting AI. They input their lyrics and specified the style they envisioned, and the AI generated a melody for them.”(Findings); “the AI reportedly served as a tool to enhance student creativity without replacing it.”(Findings) | Under exam-oriented curricula and limitations of non-music-education-specific AI tools, GenAI was used more for creative inspiration, sound experimentation, exam preparation, or project support; the teacher maintained creative boundaries through critical dialogue and by not directly accepting AI outputs. | “limited space for integration of GenAI within an examination-oriented curriculum”(Findings); “Most AI tools used were not specifically designed for music education contexts, which complicates their application.”(Findings); “While these tools could assist students in preparing for exams such as the music IGCSE examinations or project presentations, GenAI’s impact was less significant in the music classroom.”(Findings) |
| S15 | None | No direct classroom adoption decision evidence | None |  | Path 3 |  | None | None | Multisensory ecological creative environment and teacher facilitation role | “Educators are encouraged to move beyond anthropocentric models and cultivate environments where children co-create with natural materials, technologies, ambient soundscapes, and algorithmic collaborators.” (Implications); “Music educators should be supported in becoming co-researchers and facilitators of emergent, embodied creativity, rather than merely transmitting knowledge.” | Collaborative generation and stylistic exploration functions of AI music tools | “Children collaboratively used AI music tools. Their language often reflected a partnership...” / “‘We composed the lyrics together, me and the AI’” / “‘I was stuck, so I asked the AI for help, and it solved the melody problem’” / “‘Can you try a folk style for this tune?’” / “When the AI generated unexpected musical styles, a common reaction was curiosity.” (Results); “Children collaboratively used AI music tools. Their language often reflected a partnership, with statements like, ‘We composed the lyrics together, me and the AI’” (Results) | AI was used by students in classroom activities as a collaborator for lyric writing, melody generation, and stylistic exploration. | “‘We composed the lyrics together, me and the AI’” / “‘I was stuck, so I asked the AI for help, and it solved the melody problem’” / “‘Can you try a folk style for this tune?’” (Results) |
| S16 | “Initially, I believed that AI should not be integrated... However, subsequent experiences revealed...” (PMT-2 interview) | Pre-service teachers used GenAI for ideation, but rejected outputs misaligned with music expertise and teaching context, retaining teachers’ professional judgment. | “leveraged GenAI for ideation but rejected GenAI’s outputs misaligned with music expertise and teaching context.” (Discussion; author synthesis of qualitative findings) / “Pre-service music teachers retain primary responsibility for pedagogical decisions...” (Discussion; author synthesis) | Path 1; Path 3 |  |  | Improved GenAI-TPACK, self-efficacy, and critical evaluation capacity | “pre-service music teachers’ AIK, AIPK, AICK and AIPCK were significantly enhanced following the intervention” (Results; paired-samples t-test); “The six-week designing with AI intervention led to statistically significant and substantial improvements in pre-service music teachers’ self-efficacy across all four dimensions of GenAI-TPACK.” (Results) | TPACK-oriented collaborative AI-design environment | “This study implemented a six-week designing with AI intervention grounded in TPACK framework... GenAI-assisted lesson design.” (Abstract); “Pre-service music teachers employed GenAI tools to develop music lesson plans incorporating the 5E instructional model.” / “the training program was undergirded by TPACK and collective designing with AI.” (Discussion); “The 12 groups of pre-service music teachers successfully integrated the 5E model into their lesson designs while incorporating GenAI tools across diverse instructional activities...” | GenAI can generate ideas, but has hallucinations and lacks classroom/learner contextual knowledge | “Pre-service music teachers leveraged GenAI for ideation but rejected GenAI’s outputs misaligned with music expertise and teaching context.”(Discussion)/ “Pre-service music teachers retain primary responsibility for pedagogical decisions, as GenAI could hallucinate and it lacks contextual knowledge of classroom and learners.”(Discussion) | Under TPACK-oriented collaborative AI-design training and the development of critical evaluation capacity, pre-service teachers were more likely to use GenAI for ideating, evaluating, and revising lesson plans; when AI outputs did not align with music expertise or teaching context, they rejected or revised them and retained responsibility for pedagogical decisions. | “Initially, I believed that AI should not be integrated into teaching practices, as it appeared to compromise the professional dignity of teaching. However, subsequent experiences revealed that AI could indeed provide me with constructive instructional support.”(PMT-2 interview)/ “Pre-service music teachers leveraged GenAI for ideation but rejected GenAI’s outputs misaligned with music expertise and teaching context.”(Discussion; author synthesis)/ “Pre-service music teachers retain primary responsibility for pedagogical decisions...” (Discussion) |
| S17 | “Participants raised concerns about the potential biases present in AI algorithms...” (Findings) | The authors/findings recommended integrating AI through teacher training, curriculum alignment, human-centered design, and ethical frameworks. | “Educators found training... extremely valuable.” (Findings; interview-based result) / “ethical awareness and responsible AI practices are essential.” (Conclusion; author implication) | Path 4 | Path 3 | Path 1 | Knowledge and skills required for teachers’ effective AI use | “Educator training programs provide teachers with the skills and information they need to properly harness the potential of AI.” (Conclusion) / “Education professionals are kept up to date with the latest AI technologies and best practices through ongoing professional development.” (Conclusion) | Curriculum alignment, technical support, ethical standards, and privacy protection | “Problems with the network and software can stymie the learning process.” (Findings) / “These difficulties highlight the importance of strong technical support and infrastructure to guarantee that AI-based systems run smoothly.” (Discussion) / “Ethics-related factors, such as algorithm fairness and data privacy, highlight the significance of ethical AI use and the necessity of unambiguous ethical standards.” (Conclusion) / “Curriculum alignment is incorporating AI-driven resources, activities, and assessments into existing curricula on purpose.” (Discussion | Personalized feedback, real-time feedback, collaborative projects, and interface usability | “The ability of artificial intelligence to tailor courses, provide real-time feedback, and support collaborative music projects has the potential to spark student motivation, improve practice, and build excitement for music.” (Conclusion) / “User-centric design ideas and aesthetics emerge as critical concerns in AI integration.” (Conclusion) / “The adoption of intuitive user interfaces and aesthetically pleasing designs increases user pleasure and engagement...” (Conclusion) | If AI has user-centered design and is accompanied by technical support, curriculum alignment, teacher training, and ethical protection, it can be used for personalized feedback, learning support, and collaborative projects; if network, software, privacy, or ethical conditions are insufficient, integration is constrained. | “The ability of artificial intelligence to tailor courses, provide real-time feedback, and support collaborative music projects...” (Conclusion) / “Educator assistance through training programs and ongoing professional development...” (Conclusion;) / “Curriculum alignment is incorporating AI-driven resources...” (Findings) / “Problems with the network and software can stymie the learning process.” (Findings) / “These difficulties highlight the importance of strong technical support and infrastructure...” (Findings) / “ethical awareness and responsible AI practices are essential.” (Conclusion) |
| S18 | “job insecurity has a significant positive relationship with active learning intention, its mediating role... is not significant.” (mediation test) | No direct classroom adoption decision evidence | None |  | Path 3 |  | Proactive personality, job insecurity, and active learning intention | “The analysis showed the interaction term PP x JI significantly and positively influences ALI (β = 0.132, p < 0.001). This supports H5, indicating PP positively moderates the relationship between JI and ALI.” (Results) | None | None | None | None | Technology impact awareness and job insecurity were both related to active learning intention, and proactive personality strengthened the transformation from job insecurity to active learning intention. | “technology impact awareness... has a significant positive influence on music teachers’ active learning intention...” (Discussion) / “although job insecurity has a significant positive relationship with active learning intention, its mediating role between technology impact awareness and active learning intention is not significant.” (Discussion) / “PP positively moderates the relationship between JI and ALI.” (Results) |
| S19 | “teachers’ demand for algorithm transparency is increasing day by day.” (Discussion) | No direct classroom adoption decision evidence | None |  | Path 4 | Path 1 | None | None | Cross-cultural teaching demands and costs of cultural-bias correction | “In the cross-cultural classroom, teachers often have to teach music theory as well as cultural background.” (Result) / “the current cultural deviation correction mechanism is highly dependent on expert annotation, leading to high cost and slow update.” (Discussion) / “combining the strategy of community co-construction to encourage teachers and learners from different cultural backgrounds to participate in content generation and feedback is necessary...” (Discussion) | Multimodal semantic mapping, real-time feedback, dynamic demonstration, interpretable panels, and limitations in feedback latency/emotional recognition | “we put forward a multistate adaptive framework that connects the four links of data acquisition, feature alignment, interactive feedback, and teacher monitoring...” (Results) / “we set up an interpretable panel in the background to display three indicators: pitch deviation, rhythm fluctuation, and emotional matching.” (Results) / “dynamic demonstration not only optimized the speed of knowledge intake but also indirectly improved the classroom atmosphere.” (Results) / “the delay of the mobile terminal has been controlled within 180 ms, the real-time interaction in the weak network environment is still affected.” (Discussion) / “data scarcity, inaccurate identification of emotional symbols, and delay of real-time feedback...” (Abstract) | AI can be used for dynamic demonstration, auxiliary correction, and visual feedback, but cultural-bias correction, emotional-symbol recognition, algorithmic transparency, and feedback latency limit its higher-order cultural interpretation functions. | “The frequency of beat slip in the static courseware group was 23%, whereas in the intelligent group, the frequency was only 9%.” (Results) / “dynamic demonstration not only optimized the speed of knowledge intake but also indirectly improved the classroom atmosphere.” (Results) / “Teachers pay more attention to the transparency and interpret the ability of algorithms...” (Discussion) / “the current cultural deviation correction mechanism is highly dependent on expert annotation, leading to high cost and slow update.” (Discussion) |
| S20 | None | No direct classroom adoption decision evidence | None |  | Path 4 |  | None | None | None | None | Automatic music semantic retrieval, synchronous collaboration, collaborative score/recording functions, and limitations in latency and audio quality | “instructors identified several interactive features as being essential, including the possibility to adjust the playback speed of an accompaniment..., score following, audio-to-score alignment..., and audio transcription...” (User needs and key priorities) / “These priorities focus on: (1) ensuring audio quality and synchronization in music lessons, (2) enabling collaborative manipulation of sheet music, (3) supporting collaborative audio recording practices, and (4) designing semantically aware user interactions.” (User needs and key priorities) / “Audio quality issues were a major concern, with the most common problems being audio delay and synchronization...” (Survey findings) | MusiCoLab supports asynchronous and synchronous collaboration, but audio latency, synchronization, network, and user-experience issues restrict the quality and scalability of synchronous musical interaction. | “The implemented tools are aimed at both asynchronous student-teacher interactions... as well as synchronous collaborations...” (MusiCoLab approach) / “While MusiCoLab successfully integrates real-time collaboration features, its evaluation reveals the need for further refinements, in optimizing audio quality, minimizing network-related issues, and optimizing user experience.” (Conclusion) / “the study identifies key development priorities and highlights challenges such as latency, synchronization, and user experience.” (Abstract) |

**Table 4. Path Boundaries and Decision Criteria for Thematic Classification**

| Path | Core judgment question | Inclusion boundary | Exclusion boundary | Core pathway concepts | Primary-path studies | Secondary-path studies |
| --- | --- | --- | --- | --- | --- | --- |
| Path 1: Professional Boundaries and Retention of Judgment Authority | Who retains final judgment? | The original text explicitly shows that teachers, instructors, or pre-service teachers reviewed, revised, rejected, or fact-checked AI outputs, or emphasized the final role of humans in professional judgment, cultural interpretation, authorship, ethical responsibility, and pedagogical discretion. | General ethical discussion, author recommendations, or system-design assumptions about “teacher control” only; no evidence of judgment by teachers, instructors, or pre-service teachers. | Human review of AI outputs; retention of final judgment authority; rejection of outputs that are contextually inappropriate or professionally inaccurate; professional verification of AI-generated lesson plans, texts, or creative outputs. | S1, S13, S16 | S3, S7, S9, S10, S12, S17, S19 |
| Path 2: Learning-Process Regulation and Critical Engagement | How do teachers regulate the learning process? | The original text explicitly shows that teachers were concerned with the effects of AI on the learning process, original expression, critical thinking, cognitive engagement, learning depth, or classroom participation quality, and adjusted AI use accordingly. | Student achievement, learning outcomes, or system performance results only; technical descriptions of platform latency or feedback mechanisms only; no evidence of teachers’ judgment about the learning process or cognitive engagement. | Regulating the timing of AI intervention; setting rules for AI use; requiring students to think before using AI; preventing copying, misinformation, generic outputs, and overreliance; guiding critical use of AI. | S4, S9, S14 | S10 |
| Path 3: Adaptive Co-Creation and Professional Capacity Reconstruction | Does AI expand teachers’ professional capacity? | The original text explicitly shows that teachers, pre-service teachers, or educators used AI for creative generation, lesson-plan design, interactive learning, inclusive expression, professional learning, AI literacy, or GenAI-TPACK development. | Descriptions of AI potential only, without evidence of teachers’ or pre-service teachers’ use, design, learning, or capacity development; student-only AI use without teacher-related evidence is not treated as a primary pathway. | Using AI for ideation, creation, teaching-material generation, or lesson-plan design; improving AI literacy through training; using AI as a resource for creative, pedagogical, or inclusive practice. | S3, S5, S6, S16 | S1, S4, S7, S14, S15, S17, S18 |
| Path 4: Risk Perception and Bounded Adoption | What conditions shape teachers’ AI adoption? | The original text explicitly shows that AI adoption was shaped by knowledge and experience, training, resources, policy, rule frameworks, curriculum structure, institutional support, ethical/IP risks, technological accessibility, or platform conditions. | General discussion of AI advantages only; no explanation of how conditions influenced adoption intention, scope of use, integration readiness, or pedagogical boundaries. | Controlled integration when rules, training, resources, and ethical frameworks are sufficient; cautious, peripheral, or conditional adoption due to insufficient knowledge, limited resources, policy pressure, technological constraints, or unclear rules. | S2, S7, S8, S11, S12, S17 | S6, S9, S19, S20 |

**Table 5. MMAT 2018 Methodological Quality Appraisal of Empirical Studies**

| Code | Study | MMAT category | S1: Clear research questions | S2: Data address the research questions | Q1 | Q2 | Q3 | Q4 | Q5 | Overall judgment |
| --- | --- | --- | --- | --- | --- | --- | --- | --- | --- | --- |
| S2 | An Investigation of Korean Music Teachers’ Perceptions of Technology in Music Classes | Mixed methods | Y | Y | Y | CT | CT | CT | Y | Moderate |
| S4 | Design, Implementation, and Effects of Elementary Music Creation Class Using an AI-Based Music Program, Doodle Bach | Mixed methods | Y | Y | Y | Y | CT | CT | Y | Moderate-to-high |
| S5 | Do Innovative Teachers Use AI-Powered Tools More Interactively? A Study in the Context of Diffusion of Innovation Theory | Mixed methods | Y | Y | Y | CT | CT | CT | Y | Moderate |
| S6 | Educational Equity at the Geopolitical Frontier: A Music-Based Teacher Training Case | Qualitative | Y | Y | Y | Y | Y | Y | CT | High |
| S7 | Empowering Faculty in Creative and Cultural Disciplines: AI Literacy and Image Generator Integration in Higher Education | Mixed methods | Y | Y | Y | Y | Y | Y | Y | High |
| S8 | Exploring Pre-Service Music Teachers’ Acceptance of Generative Artificial Intelligence: A PLS-SEM-ANN Approach | Quantitative descriptive | Y | Y | Y | CT | Y | CT | Y | Moderate-to-high |
| S9 | Exploring Teachers’ Beliefs About ChatGPT in Arts Education | Qualitative | Y | Y | Y | Y | Y | Y | Y | High |
| S10 | Integrating ChatGPT for Music History Education | Mixed methods | Y | Y | CT | CT | CT | CT | Y | Low-to-moderate |
| S11 | Modeling Music Student Teachers’ Behavioral Intention of Using Artificial Intelligence in China | Quantitative descriptive | Y | Y | Y | CT | Y | CT | Y | Moderate-to-high |
| S13 | Music Teachers’ Labeling Accuracy and Quality Ratings of Lesson Plans by Artificial Intelligence (AI) and Humans | Quantitative descriptive | Y | Y | Y | CT | Y | CT | Y | Moderate-to-high |
| S14 | Navigating the Intersection of Generative Artificial Intelligence and Democratic Pedagogy in Music Education in Macau | Qualitative | Y | Y | Y | Y | Y | Y | CT | High |
| S15 | Posthumanism in Cultivating Children’s Musical Creative Practice: A Theoretical Framework and Case Study from a Primary School | Qualitative | Y | Y | Y | Y | Y | Y | CT | High |
| S16 | Promoting Pre-Service Music Teachers’ TPACK with Generative AI: An Intervention Through Designing with AI | Mixed methods | Y | Y | Y | Y | Y | Y | Y | High |
| S17 | Research on Human-Centered Design in College Music Education to Improve Student Experience of Artificial Intelligence-Based Information Systems | Qualitative | Y | Y | Y | Y | Y | Y | CT | High |
| S18 | Resistance or Resilience? University Music Teachers’ Active Learning Intention in Response to the Emergence of Artificial Intelligence | Quantitative descriptive | Y | Y | Y | CT | Y | CT | Y | Moderate-to-high |
| S19 | The Auxiliary Function and Realization Mechanism of Artificial Intelligence in Cross-Cultural Traditional Music Education | Quantitative non-randomized | Y | Y | CT | Y | CT | CT | Y | Moderate |
| S20 | Web-Based Collaborative Music Lessons: Approaches, Challenges and Perspectives | Quantitative descriptive | Y | Y | CT | CT | CT | CT | Y | Low-to-moderate |
